# The Impact of Milk on Gut Permeability, Fecal 16S rRNA Gene Microbiota Profiling, and Fecal Metabolomics in Children with Moderate Malnutrition in Sierra Leone: A Double-Blind, Randomized Controlled Trial

**DOI:** 10.1016/j.ajcnut.2024.09.018

**Published:** 2024-09-21

**Authors:** Minsoo Son, Marie L Laury, Kevin B Stephenson, Thaddaeus May, D Taylor Hendrixson, Aminata Shamit Koroma, Amara Stevens Ngegbai, Jong Hee Song, Nino Naskidashvili, Young Ah Goo, Mark J Manary

**Affiliations:** 1 Mass Spectrometry Technology Access Center at the McDonnell Genome Institute, Washington University, St. Louis, MO, United States; 2 Genome Technology Access Center, McDonnell Genome Institute, Washington University, St. Louis, MO, United States; 3 Department of Internal Medicine, Washington University, St. Louis, MO, United States; 4 Department of Internal Medicine, Baylor College of Medicine, Houston, TX, United States; 5 Department of Pediatrics, University of Washington, Seattle, WA, United States; 6 Ministry of Health, The Republic of Sierra Leone, Freetown, Sierra Leon; 7 Brown School, Washington University, St. Louis, MO, United States; 8 Department of Pediatrics, Washington University School of Medicine, One Children’s Place, Saint Louis, MO, United States; 9 Children’s Nutrition Research Center, Baylor College of Medicine, Houston, TX, United States

**Keywords:** milk, moderate malnutrition, gut health, EED, microbiome

## Abstract

**Background:**

Bovine milk is a beneficial ingredient in teh treatment of malnutrition.

**Objectives:**

Our objectives were to determine the effect of dietary milk protein and milk carbohydrate on the intestinal permeability, fecal 16S rRNA gene configuration, and fecal metabolomics of children with moderate malnutrition.

**Methods:**

This was a randomized, double-blind, controlled trial among 413 children with wasting in rural Sierra Leone who received 1 of the following 4 supplementary foods, which differed in sources of protein and carbohydrate: milk protein and milk carbohydrate (MPMC), milk protein and vegetable carbohydrate (MPVC), vegetable protein and milk carbohydrate (VPMC), or a control group consuming entirely vegetable-based food (VPVC). After 4 wk, urine and stool were collected from participants enrolled with mid-upper arm circumference of <12.1 cm. Urine was analyzed for lactulose excretion (%L). Stool samples were subjected to both 16S rRNA gene analysis to assess β-diversity and untargeted metabolomic abundance.

**Results:**

Among the 386 children who completed permeability testing, the mean difference (95% CI) in %L excretion as compared with VPVC was 0.01 (−0.05, 0.07) for MPMC, 0.05 (−0.01, 0.11) for MPVC, and 0.01 (−0.05, 0.07) for VPMC. Of the 374 children who provided a stool sample that was analyzed, the β-diversity among bacterial taxa was similar between dietary groups (*P* > 0.05 for all comparisons). No significant differences between dietary groups were seen among the 20 most abundant bacterial taxa. Among the 5769 unique metabolomic features identified, greater flavonoid levels in VPVC were seen.

**Conclusions:**

Abnormal intestinal permeability do not improve with 4 wk of supplementary feeding. Fecal rRNA do not differ with consumption of different diets.

This trial was registered at clinicaltrials.gov as NCT04216043.

## Introduction

Annually, there are >160 million children identified with acute malnutrition. The WHO recommends that children with severe acute malnutrition (SAM) receive a therapeutic food in which half of the protein is provided from dairy sources [1]. The basis of this recommendation is that children with malnutrition who receive more dairy protein have superior clinical outcomes [2]. Even for children with moderate acute malnutrition (MAM), supplementary food containing milk powder increases rates of weight gain and recovery [3]. Skimmed milk powder consists largely of milk protein (MP) and lactose and is the primary dairy ingredient used in food aid products. It is not known whether the clinical benefits seen in children with acute malnutrition are the result of MP, lactose, or both.

MP is considered an ideal dietary protein for children because it is rich in essential amino acids that are highly digestible. Children with acute malnutrition need to rapidly rebuild metabolically active tissues, including their gut epithelium. The gut epithelium is an important barrier between the environment and the individual and is the site where ingested nutrients are absorbed. Children with acute malnutrition are at greater risk of environmental enteric dysfunction (EED), an inflammatory condition of the small bowel characterized by increased permeability, inflammatory cell infiltrate, and decreased absorptive surface area [4]. It is possible that MP facilitates the recovery of the small bowel and reduces EED in acute wasting better than vegetable protein (VP) and that this may be the mechanism whereby milk yields better clinical outcomes.

Lactose is the primary carbohydrate in MP, comprising 56% of its content. Lactose is a disaccharide that is often digested less efficiently than other sugars in the human intestinal tract. In acute malnutrition, synthesis of brush border disaccharidases that line the gut epithelium is reduced and lactose absorption suffers as a consequence. Unabsorbed lactose has even been speculated to contribute to malabsorption in children with malnutrition [5]. Unabsorbed carbohydrates also have the potential to support gut microbial growth, and hence fermentative production of essential nutrients. Although the duodenum and jejunum contain limited numbers of microbes, the ileum is home to the greatest numbers of bacteria in the human intestine. The ileum has a similar capability to absorb nutrients as the duodenum and jejunum. It is possible that lactose leads to altered microbial populations and metabolic activities that might explain in part why milk products yield superior clinical outcomes in children with malnutrition.

To better understand the roles of MP and lactose in the diets of children with MAM, we conducted a clinical trial in which children with MAM received 1 of the 4 supplementary foods that differed in protein and carbohydrate sources: milk protein and milk carbohydrate (MPMC), milk protein and vegetable carbohydrate (VC; MPVC), soy protein (VP) and milk carbohydrate (VPMC), or soy (VPVC). We tested the following hypotheses: *1*) children receiving MPMC or MPVC would demonstrate reduced intestinal permeability on a lactulose excretion test and *2*) children receiving MPMC or VPMC would demonstrate greater β-diversity in their fecal 16S rRNA gene configuration, with greater β-diversity.

## Methods

### Study design

This prospective, randomized, double-blind, controlled clinical trial compared the effects of 4 ready-to-use, peanut-based supplementary foods for treatment of children with MAM. The primary objective was to assess the impact of the study foods on gut permeability and the configuration of the fecal microbiome as 2 potential mechanisms underlying the clinical benefit of milk-containing supplementary foods for the treatment of children with MAM. The primary outcomes were percent of lactulose excretion (%L) and 16S rRNA gene β-diversity. The original trial registration erroneously identified the primary outcomes as recovery and weight gain. The trial was not designed or powered to detect changes in recovery or weight gain, which were secondary outcomes. This error was corrected before submission of the article. Recovery and weight gain are reported.

**FIGURE 1:**
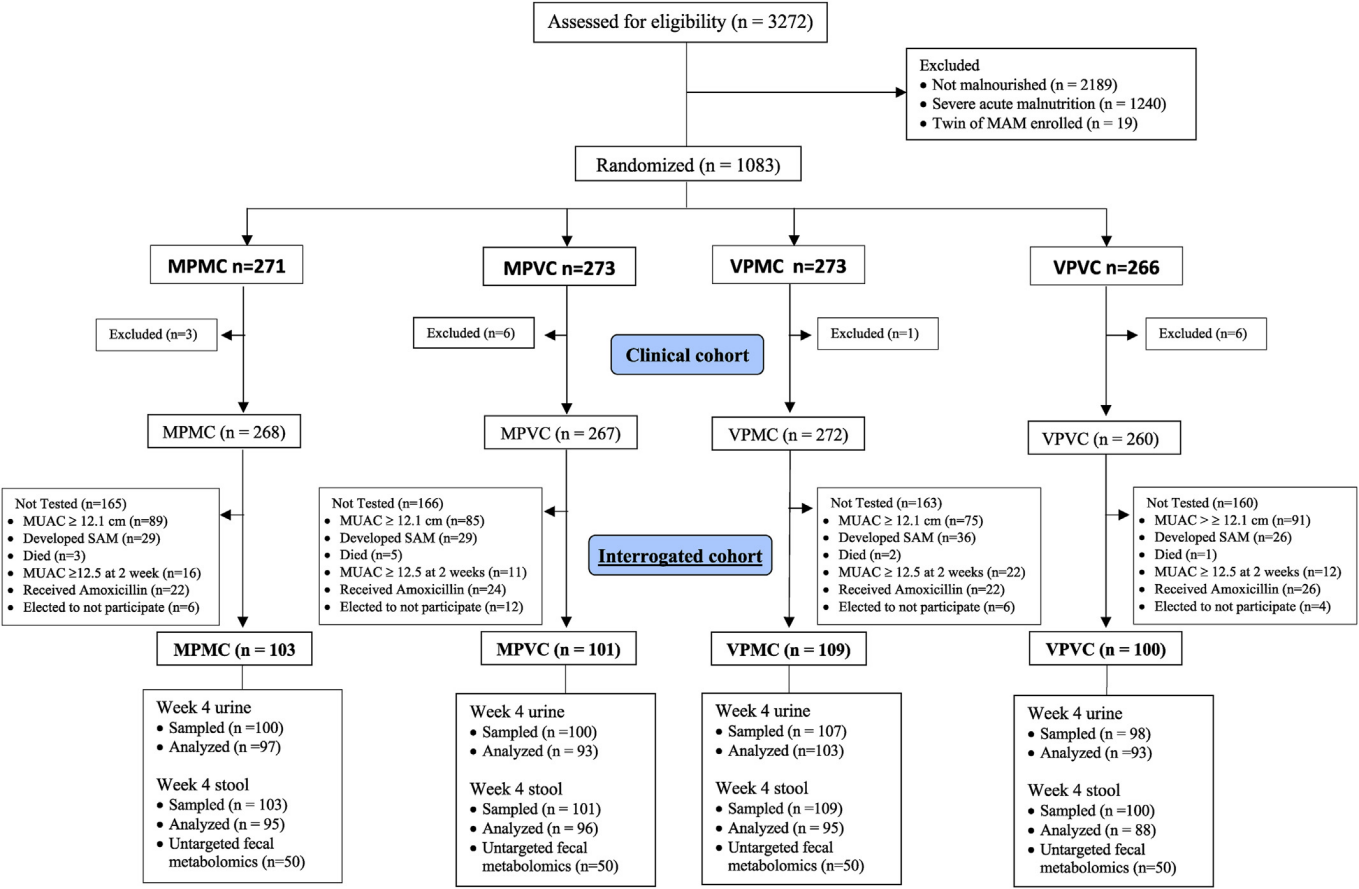
Consort substudy participant flow diagram. MAM, moderate acute malnutrition; MPMC, milk protein/milk carbohydrate; MPVC, milk protein/vegetable carbohydrate; VPMC, vegetable protein/milk carbohydrate; VPVC, vegetable protein/vegetable carbohydrate.

The trial consisted of the following 3 nested groups: *1*) the largest group (clinical cohort) including all children enrolled and randomly assigned on the basis of MAM diagnosis, with mid-upper arm circumference (MUAC) of 11.5–12.4 cm; *2*) a subset of these children (sampled cohort) with enrollment MUAC of <12.1 cm who underwent sample collection for assessment of intestinal permeability and fecal microbiome; and *3*) a further subset of the sampled cohort who underwent fecal metabolome analysis (Figure 1). Fecal and urine sampling was restricted to children with enrollment MUAC of <12.1 cm. This was done in order to enrich the sampled cohort for more severe MAM and to avoid studying participants in whom the diagnosis of acute malnutrition is less certain. To further explicate the latter point, MUAC is an absolute value and is not adjusted for age or sex. When MUAC values are compared against age-based and sex-based global norms, the distribution of MUAC values for younger girls is lower than that for boys and older girls, irrespective of nutrition status [6]. The alternative method for diagnosing MAM relies on weight-for-length *z*-scores, which are sex-adjusted and compared against a global distribution. For weight-for-length *z*-scores, a cutoff of 2 *z*-scores below the mean is used to diagnose MAM. An MUAC of 12.4 cm is technically diagnostic of MAM, but when evaluated using MUAC *z*-scores, a value of 12.4 cm does not fall below 2 *z*-scores below average until a girl exceeds 84 wk of age, which is 30 wk older than the average age at time of MAM diagnosis in rural Sierra Leone. Because this study was designed to assess potential mechanisms whereby MP or MC confer their benefits to children with acute malnutrition, we restricted sampling to those in whom acute malnutrition was a more certain diagnosis. The target sample size was determined on the basis of the sampled cohort and using the coprimary outcome, %L excretion, as this required more participants than 16S rRNA β-diversity. In a previous study in the same district of Sierra Leone among children with malnutrition, mean %L excretion was 0.32% (SD = 0.3%) after 4 wk of treatment [7]. To detect a mean difference in lactulose excretion of 0.13%, a 40% relative reduction and one which would be sufficient to bring the average participant below a commonly used EED threshold of 0.2, it was estimated that a sample size of 90 participants per group would provide 80% power at a 2-sided α of 0.05 [8]. Regarding the fecal microbiome primary outcome, we previously detected fecal microbiota differences in 81 children with MAM and EED, thus representing a smaller sample size requirement than %L [9]. To account for ≤5% samples that may not be adequate for analysis or lost, we targeted a conservative total enrollment of 380 posttreatment analyses. This represents the target sample size for the sampled cohort, in which the primary outcomes were to be assessed. Based on previous clinical trial data on children with MAM in Sierra Leone, it was estimated that 1000 total participants would be required for enrollment into the clinical cohort to achieve this number. This accounts for 32% of participants with MUAC of ≥12.1 cm, 20% deteriorate to SAM before sample collection, and the 10% defaulting before sample collection.

The study was approved by the Human Research Protection Office at Washington University (ID# 201912091) and by the Sierra Leone Ethics and Scientific Review Committee (no registration/approval number assigned, filed under the title “Milk matters in malnutrition, is it the lactose or dairy protein?”). This trial was registered at clinicaltrials.gov as NCT04216043 (https://clinicaltrials.gov/study/NCT04216043?id=NCT04216043&rank=1).

### Study population

Subjects were enrolled from 16 September, 2020, through 11 November, 2021, in Pujehun, a rural district in Sierra Leone. Children were followed up for a period of 4–6 wk after enrollment. Participants were enrolled from 17 peripheral health units where fortnightly malnutrition clinics were held. Children were eligible for enrollment if they were between the ages of 6 and 59 mo and were diagnosed with MAM as defined by MUAC >11.4 cm and <12.5 cm and the absence of bilateral pedal pitting edema. To be eligible for urine and stool collection (sample cohort), children had to have enrollment MUAC of <12.1 cm. Children with chronic medical conditions, such as cerebral palsy, tuberculosis, or HIV and those with a history of peanut or milk allergy or who had received treatment of malnutrition in the preceding 28 days were excluded. Informed consent was obtained from the primary caregiver and documented by a signature or thumbprint if the caregiver was unable to write.

### Randomization and blinding

Study foods were similar in appearance and texture and were packaged in identical foil sachets, except for the presence of a colored sticker which corresponded to food group. An individual who was not otherwise engaged with the trial produced the key linking color to food. Following production, research staff applied the colored labels to the food packets and boxes; these research staff were not engaged in the study otherwise, including participant enrollment, outcomes assessment, or food distribution. Field and laboratory staff were blinded to the allocation code. Investigators responsible for statistical analyses remained blinded until analyses were complete, except when comparisons were made between combinations of groups, for example, when comparing the 2 groups with MP with the 2 with VP, as identification of which groups shared common macronutrient sources was necessary for these analyses.

Randomization was done by participants. Before trial initiation, a research team member prepared blocks of 20 small opaque envelopes. Within each block, there would be 5 envelopes made for allocation to each study group by placing within each envelope a small piece of colored paper matching the group’s color. Each block of 20 small opaque envelopes would then be shuffled manually and put into a larger opaque envelope. Participants were randomly assigned when their caregiver would reach into a larger opaque envelope without looking and remove a single small opaque envelope, which they would then open to reveal the colored piece of paper within, thereby allocating the participant to their group.

### Participation

Upon enrollment, socioeconomic information, health histories, and anthropometric data were collected. A convenience subset of 50 participants per group with enrollment MUAC of <12.1 cm were invited to undergo urine and stool collection at enrollment. All participants with enrollment MUAC of <12.1 cm were notified of the intention to perform intestinal permeability testing and collect stool after 4 wk of feeding. Participants returned for fortnightly visits, during which anthropometry was measured and a report of symptoms collected. Fourteen packets of food were dispensed at each visit. If a participant was determined to have deteriorated to SAM at a follow-up visit, the study food was stopped and ready-to-use therapeutic food and amoxicillin were dispensed according to the local standard of care. If a participant’s MUAC was found to have increased to >12.4 cm, they were deemed to have recovered from MAM and feeding was discontinued. After 4 weeks of treatment, all children who had been enrolled with an MUAC of <12.1 cm were invited to participate in permeability testing and fecal specimen collection.

### Study foods

Four isoenergetic foods were created for this study (Table 1). These foods differed in that either MP or VP was combined with either MC or VC, with some resulting variation required in peanut and sugar (sucrose) amounts to ensure similarity in caloric density, total protein provision, texture, and taste. Skimmed milk powder was used in MPMC, whey protein isolate with maize flour in MPVC, soy flour and whey permeate in VPMC, and soy and maize in VPVC. Study foods were produced by Project Peanut Butter, a local producer of food aid products in Sierra Leone. Appropriate feeding technique was demonstrated to each participant’s caregiver at each visit by trained research nurses. Research staff also emphasized that the study food was meant for the child with MAM and was not to be shared with others.

**TABLE 1:** Composition of supplements provided to children with moderate acute malnutrition

Ingredient	Milk protein milkcarbohydrate	Milk protein vegetablecarbohydrate	Vegetable proteinmilk carbohydrate	Vegetable proteinvegetable carbohydrate
Peanuts (g/100 g)	23.7	15.2	9.1	9.1
Canola oil (g/100 g)	5.1	26	25.5	26
Palm oil (g/100 g)	16.0	1	1	1
Maize flour (g/100 g)	0	18.4	0	10
Sugar (g/100 g)	23.0	23	9	16
Soy flour (g/100 g)	0	0	33	33
Skim milk powder (g/100 g)	27.3	0	0	0
Whey protein isolate (g/100 g)	0	11.5	0	0
Whey permeate (g/100 g)	0	0	17.5	0
Vitamin premix (g/100 g)^1^	2.9	2.9	2.9	2.9
Hydrogenated soy oil (g/100 g)	2.0	2.0	2.0	2.0
Milk protein (g/100 g)	9.8	10.6	0	0
Lactose (g/100 g)	13.8	0.1	14.9	0
Protein (%)	15.8	15.4	15.2	15.5
Energy (kcal/100 g)	552	550	548	551

1 The micronutrient premix consists of 14 vitamins and minerals in dosage that provide about 2× the Recommended Daily Alowance, suitable for treating severe wasting.

### Intestinal permeability, fecal microbiome, and fecal metabolome determinations

Intestinal permeability testing and fecal collection were conducted as previously described [10]. Briefly, lactulose permeability (%L) was measured after the child consumed 5 g of lactulose dissolved in 20 mL of water, followed by a complete 4-h urine collection. The total volume of collected urine was recorded. Urine was preserved with 10 mg merthiolate before flash freezing in liquid nitrogen in the field. Thereafter, urine was stored at −80°C until analyses. Urine was analyzed by HPLC. %L was categorized as follows: normal, %L < 0.20; increased, %L ≥ 0.20% and <0.45%; and markedly increased, %L ≥ 0.45% [11].

Fecal specimens were collected fresh, flash frozen in liquid nitrogen within 5 minutes of passing, and subsequently stored at −80°C until analyses. Genomic DNA was extracted, following the manufacturer’s instructions, using the QIAmp PowerFecal pro DNA kit (Qiagen) automated in the QIAcube.

Samples for fecal microbiome were amplified using a targeted DNA sequencing library preparation using the Standard Biotools System (standardbio.com). This method used an integrated microfluidics chip, which simultaneously amplifies several amplicons that are indexed to each sample. The pool of samples were cleaned to remove excess primers and then the Ilumina sequence adapters were added by a PCR. The pool was further cleaned using bead purification, quality controlled using measurement of nucleic acid content, and loaded onto an appropriate Illumina sequencer [12]. The MVRSION pipeline was used for taxonomic assignment, which was made with QIIME. QIIME2 was used for diversity analyses. Demultiplexed reads from seven 16S rRNA gene amplicons covering 8 of the 9 variable regions were analyzed using the MVRSION pipeline to generate a list of microbial species with their corresponding number of reads for each sample [12]. Default parameters were used for the MVRSION analysis in conjunction with the Silva 132 database, and data were rarefied to a depth of 1500 reads.

In addition to the 16S rRNA gene sequencing described for the entire sample set, a convenience subset of 168 fecal 16S rRNA gene samples were further sequenced at ∼20-fold greater depth, in an effort to determine whether this would yield different β-diversity scores or uncover differences in the number of taxa between the food groups. The intention was to assess the adequacy of the depth of sequencing. The convenience subset included all of our tested samples from 2023 and were not selected using any other criteria.

The fecal metabolites were extracted from 65 mg feces from each subject by 2 separate extraction methods, one with water for aqueous extraction and the other with dichloromethane for organic extraction [13]. Both extraction processes included methanol as a second solvent and zirconium bead beating. Extracted metabolites were analyzed on Orbitrap ID-X Tribrid Mass Spectrometer (ThermoFisher Scientific) equipped with Vanquish Horizon UHPLC (ThermoFisher Scientific). The following 4 independent liquid chromatography tandem mass spectrometry (LC-MS/MS) methods were used to detect and measure complementary classes of metabolites: *1*) Hydrophilic interaction liquid chromatography column in positive mode for positive polar metabolites [14]; *2*) C18 column in positive mode for intermediate polar metabolites [15]; *3*) amide column with high-pH mobile phases in a negative mode for negative polar metabolites [16]; and *4*) C8 column in positive mode for lipids [17]. Aqueous extracts were analyzed with the first 3 methods and organic extracts were analyzed with the last method. In total, 800 runs of LC-MS/MS data from 200 fecal samples from 2 extraction methods were processed by Compound Discoverer 3.3 (ThermoFisher Scientific) for compound annotations. The methods for LC-MS analyses and data acquisition are detailed in Supplemental Methods.

### Outcomes

With 1 exception, all outcomes were compared between milk-containing supplementary food groups and the all-vegetable group: MPMC compared with VPVC, MPVC compared with VPVC, and VPMC compared with VPVC. The primary outcomes were %L and weighted Uni-Frac differences between fecal 16S rRNA gene configurations as a measure of β-diversity. Key secondary outcomes were fecal 16S rRNA gene relative abundances and the metabolic feature abundance, which was compared in a different manner: children receiving MP compared with those receiving VP, children receiving MC compared with those receiving VC, and children receiving any milk-containing product (MPMC, MPVC, and VPMC) compared with those receiving VPVC. Other secondary outcomes were α-diversity of microbiome taxa, differences in metabolic feature abundance between dietary groups, rate of change in weight, length, and MUAC, recovery, and deterioration to SAM.

### Statistical analyses

Clinical data were double entered and checked for discrepancies, which were resolved by examination of the primary data cards. Anthropometric indices were calculated using WHO 2006 Child Growth Standards (Anthro version 3.2.2) [6]. Categorical variables were summarized using number (percentages), whereas continuous variables were summarized using mean ± SD if their distribution visually approximated normality or median (IQR) if not. Modified intention-to-treat analysis was performed, wherein participants who were discovered to have an exclusion criterion after allocation were excluded from analysis, as were those for whom outcome data were not available. No missing data were imputed. For the comparisons of %L and β-diversity of fecal microbiome, *P* < 0.05 was considered statistically significant.

%L and 16S rRNA gene configuration data from children receiving MPMC, MPVC, and VPMC were compared with those receiving VPVC. %L was analyzed as a continuous variable using the Wilcoxon rank-sum test; pseudomedians and nonparametric bootstrapped 95% CIs were estimated. The distribution of permeability values was visualized using a ridgeline plot [18]. Characterizations of the β-diversity of the 16S rRNA gene community structures were made using weighted UniFrac distances. The dietary groups were compared using PERMANOVA, a multivariate ANOVA with permutations. α-Diversity was estimated by the Shannon index distributions within each intervention group. These were compared using the Wilcoxon rank-sum test with pseudomedians and nonparametric bootstrapped 95% CIs and visualized using a ridgeline plot.

Among participants with samples taken both at baseline and week 4, changes in %L and the Shannon index were compared across groups using linear regression, with change in the outcome as the dependent variable, the baseline value as the covariate, and dietary group as the independent variable of interest. Homoscedasticity and normality of residuals were confirmed before linear regression implementation.

A complete list of metabolic features was compiled from the 4 modes of LC-MS/MS analyzes. Features with a signal lower than the background noise, those containing large data gaps, those with peaks that were early eluting, broad or irregularly shaped, and those identified as invalid after quality control sorting were removed from the list. For the listed features, data were normalized for batch effect by locally estimated scatterplot smoothing signal correction [19] and for injection amount using a standard Centering program [20]. This comprehensive feature list contains a continuous, numerical value between −1 and 1 for the signal strength for every clinical sample analyzed, a measure of the quantity of that feature.

Comparisons of feature quantities between 2 groups were made using Student *t* test, whereas comparisons of feature quantities between >2 groups were made using ANOVA. The false discovery rate (FDR) was estimated using the Benjamini–Hochberg procedure. The generalized linear model was used to generate *t* statistics. The correlation between metabolic features and bacteria was tested by the Spearman correlation. Wilcoxon signed-rank test was performed to identify enrichment of compound class by individual metabolic features in corresponding class or to identify associations between genus and compound classes. Metabolomic features with a *P* < 0.05 and FDR < 0.05 were considered to be statistically significant [21]. The number of follow-up visits required for graduation was compared using Wilcoxon rank-sum test, whereas the Hodges–Lehmann estimator was used to generate the median of the differences between groups as well as 95% CI. Categorical outcomes including recovery were compared between groups using modified Poisson regression with robust variance estimates.

## Results

Of the 1067 children enrolled and fed (Supplemental Figure 1), 413 had an MUAC of ≤12.1 cm at baseline and had specimens of stool and urine collected at the 4-wk visit to assess the study’s primary outcome measures (Figure 1). At enrollment, participants were a median 11.7 mo old, 57% were female sex, >75% were still lactating, 70% were stunted, and the median enrollment MUAC was 11.8 cm (Table 2).

**TABLE 2:** Selected baseline characteristics of Sierra Leonian children with moderate wasting who received supplementary foods with different milk and vegetable content^1^

Characteristic	Milk protein Milkcarbohydrate (*n* = 268)	Milk protein vegetablecarbohydrate (*n* = 267)	Vegetable protein milkcarbohydrate (*n* = 272)	Vegetable protein vegetablecarbohydrate (*n* = 260)
Age (mo), median (IQR)	11.4 (8.4, 17.0)	11.6 (8.0, 17.0)	11.7 (8.1, 17.6)	11.9 (8.3, 17.2)
Female sex	158 (59)	146 (55)	152 (56)	158 (61)
Lactating	206 (77)	210 (79)	213 (78)	192 (74)
Mother is primary caregiver	245 (91)	249 (93)	248 (91)	235 (90)
Father in the home	179 (67)	209 (78)	202 (74)	193 (74)
No. of siblings	1.8 ± 1.7	1.8 ± 1.7	1.7 ± 1.7	1.8 ± 1.8
Metallic roof	226 (84)	225 (84)	233 (86)	223 (86)
Household owns motorbike	64 (24)	71 (27)	70 (26)	73 (28)
Animals sleep in the home	39 (15)	36 (13)	45 (17)	39 (15)
Water drawn from river or stream	34 (13)	26 (9.7)	26 (9.6)	33 (13)
Anthropometry				
MUAC (cm), median (IQR)	11.8 (11.7, 12.1)	11.8 (11.7, 12.1)	11.8 (11.6, 12.0)	11.9 (11.7, 12.1)
Weight (kg)	6.65 ± 0.88	6.74 ± 0.88	6.71 ± 0.92	6.68 ± 0.87
Length (cm)	68.8 ± 5.6	68.9 ± 5.4	69.1 ± 5.7	68.8 ± 5.6
LAZ	−2.6 ± 1.3	−2.6 ± 1.1	−2.5 ± 1.2	−2.7 ± 1.4
LAZ < −2	188 (70)	187 (70)	179 (66)	187 (72)
Weight-for-length *z*-score	−2.1 ± 0.7	−2.0 ± 0.8	−2.1 ± 0.8	−2.1 ± 0.9
Weight-for-age *z*-score	−3.0 ± 0.8	−2.9 ± 0.8	−2.9 ± 0.8	−3.0 ± 0.9
MUAC-for-age *z*-score	−2.4 ± 0.5	−2.5 ± 0.5	−2.5 ± 0.4	−2.4 ± 0.5
Symptoms reported in previous 2 wk				
Fever	126 (47)	143 (54)	139 (51)	124 (48)
Diarrhea	37 (14)	29 (11)	31 (11)	24 (9.2)
Cough	96 (36)	107 (40)	104 (38)	91 (35)

MUAC, mid-upper arm circumference; LAZ, length-for-age *z*-score.

1 Values are mean ± SD or *n* (%) unless otherwise indicated.

**FIGURE 2:**
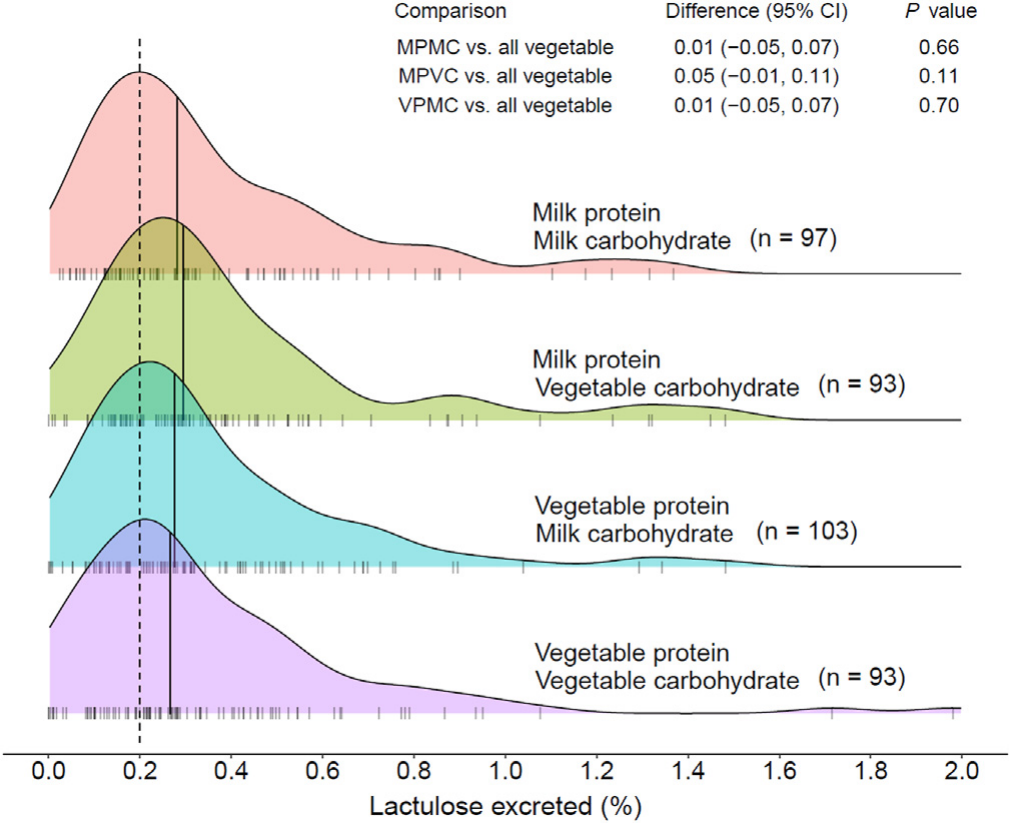
Intestinal permeability measured as % lactulose (%L) 4 wk after the onset of supplementary feeding. Normal permeability is %L of <0.20 indicated by the dashed line. Comparisons made using the Wilcoxon rank-sum test with estimated pseudomedians and nonparametric bootstrapped 95% CIs. The sample size numbers indicate the numbers of children from whom a complete 4-h urine collection was obtained and the lactulose concentration plausible.

There were no differences in %L between intervention groups after 4 wk of supplementary feeding (Figure 2). As compared with VPVC, the mean difference in %L for MPMC was 0.01 (95% CI: −0.05, 0.07; *P* = 0.66), for MPVC was 0.05 (95% CI: −0.01, 0.11; *P* = 0.11), and for VPMC was 0.01 (95% CI: −0.05, 0.07; *P* = 0.70). Abnormal intestinal permeability, defined as %L of ≥0.2, was present in 125 of the 198 (63%) of participants at enrollment and 271 of the 393 (69%) of participants after 4 wk of supplementary feeding. Among the 131 participants who underwent intestinal permeability testing at baseline and after 4 wk of feeding, no trend to decreasing %L was observed (Supplemental Table 1, Supplemental Figure 2).

**FIGURE 3:**
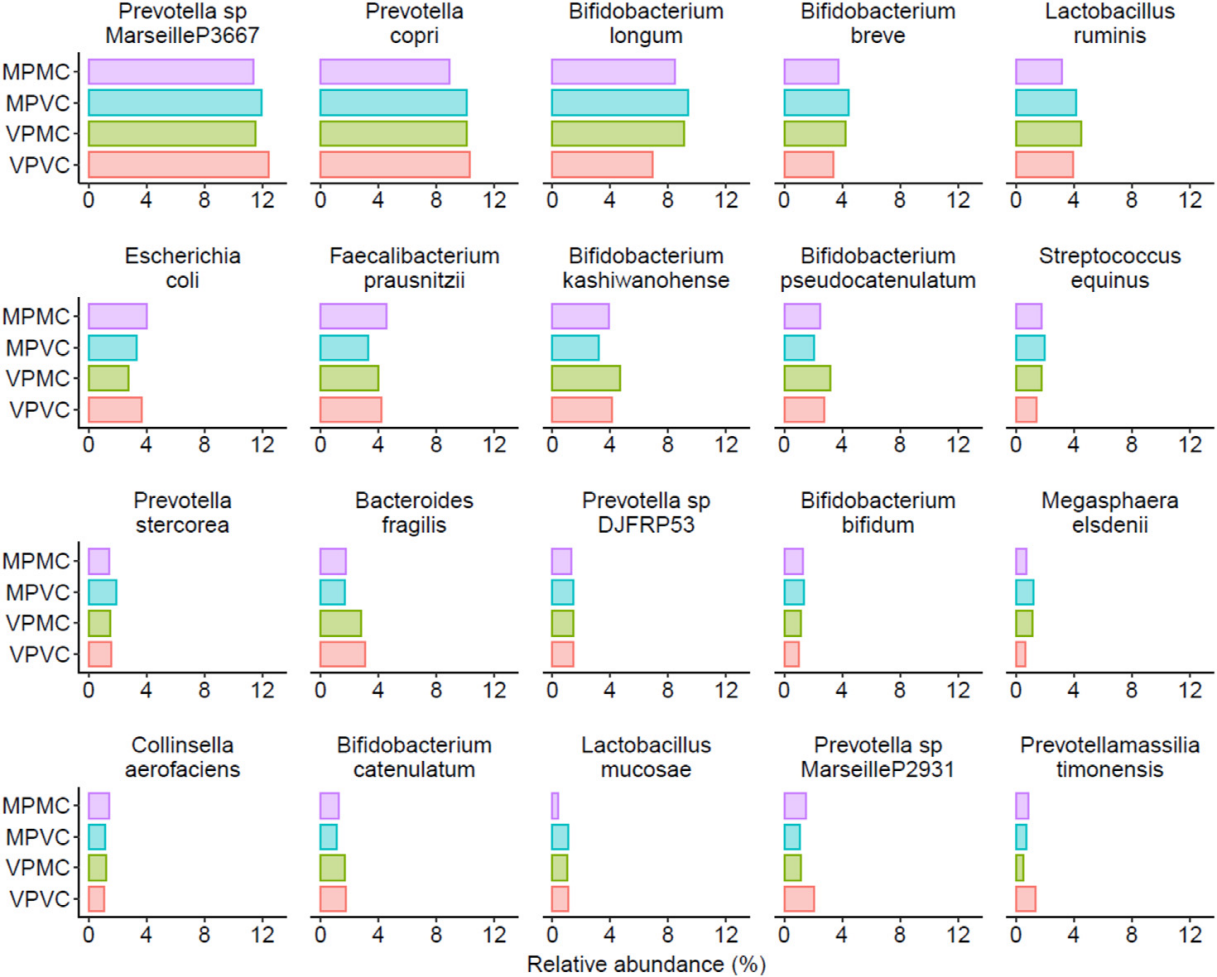
Median relative abundance of the 20 most abundant organisms detected via 16S rRNA gene analysis among Sierra Leonean children with high-risk moderate wasting who received 4 wk of supplementary feeding with study foods containing varying milk and vegetable protein and carbohydrate content. Milk protein milk carbohydrate (MPMC, *n* = 95), milk protein vegetable carbohydrate (MPVC, *n* = 96), vegetable protein milk carbohydrate (VPMC, *n* = 95), and vegetable protein vegetable carbohydrate (VPVC, *n* = 88). The median is indicated by the vertical line, the colored bar shows the 25th percentile to 75th percentile, and gray line shows the extent of 1.5× (intraquartile range). Outliers are depicted as circles. Fifty-six extreme outliers are not depicted, which had a relative abundance of >30%. The MVRSION pipeline was used to analyze demultiplexed reads from the 7 amplicons covering 8 variable regions to generate a list of microbial species with their corresponding number of reads in each sample [12]. Relative abundances were computed by dividing the absolute abundance of each species by the total species abundance in the sample. Statistical testing was performed on absolute abundance using the Wilcoxon rank-sum test with a false discovery rate of <0.10 using the Benjamini–Hochberg method.

The median (IQR) number of 16S rRNA gene reads was 34,669 (30,806–37,847) per fecal sample. The weighted UniFrac differences for MPMC (0.182; *P* = 0.51), MPVC (0.629; *P* = 0.54), and VPMC (0.181; *P* = 0.43) are as listed. Thus, when compared with VPVC, no significant differences in β-diversity with any diets containing milk were found. α-Diversity as measured by the Shannon index was similar between the 4 dietary groups as well (Supplemental Table 2 and Supplemental Figures 3 and 4). As compared with VPVC, the mean difference in the Shannon index for MPMC was 0.11 (95% CI: −0.07, 0.29; *P* = 0.23), for MPVC was 0.02 (95% CI: −0.15, 0.19; *P* = 0.79), and for VPMC was 0.03 (95% CI: −0.14, 0.18; *P* = 0.66). To visually demonstrate the similarity of the fecal 16S rRNA gene distributions the 20 most prevalent fecal species were considered. No significant differences in median relative abundance were detected between the 4 dietary groups (Figure 3).

Among the 168 specimens that were additionally interrogated using the deeper 16S rRNA gene sequencing multiamplicon methodology, the median number of reads per specimen was 22.5-fold greater than standard multiamplicon sequencing: 751,296 reads per specimen. Standard multiamplicon sequencing identified 432 species, whereas deep multiamplicon sequencing identified 414 species. The average number of species per specimen identified using standard multiamplicon sequencing was 56, whereas deep sequencing identified 51. Of the 168 specimens that underwent both sequencing depth analyses, standard sequencing revealed 6 to have <7500 reads. For these 6 specimens, deeper sequencing identified more taxa: 43 per specimen compared with 16 per specimen. For the remaining 162 specimens, standard multiamplicon sequencing identified on average 6 more taxa than deep sequencing. The relative abundances of the most prevalent 20 taxa identified by deep sequencing were similar as what was found using standard sequencing (Supplemental Figure 5). The comparisons of α-diversity and β-diversity among taxa identified by deep sequencing 16S rRNA gene were similar to that found by standard 16S rRNA gene sequencing (Supplemental Figure 6).

Using data from the 168 specimens that were interrogated with 16S rRNA gene deep sequencing, a regression model aiming to explain variation in Shannon diversity was created with 26 demographic, clinical, and dietary variables. The unadjusted *r*^2^ for the model was 0.24. The variables that were contributed most to the model were month of enrollment (partial *r*^2^ = 10%), location of child’s residence (partial *r*^2^ = 7%), and child’s age (partial *r*^2^ = 3.6%). Food group contributed <0.2%. The details and results of this modeling are presented in Supplemental Table 3.

**FIGURE 4:**
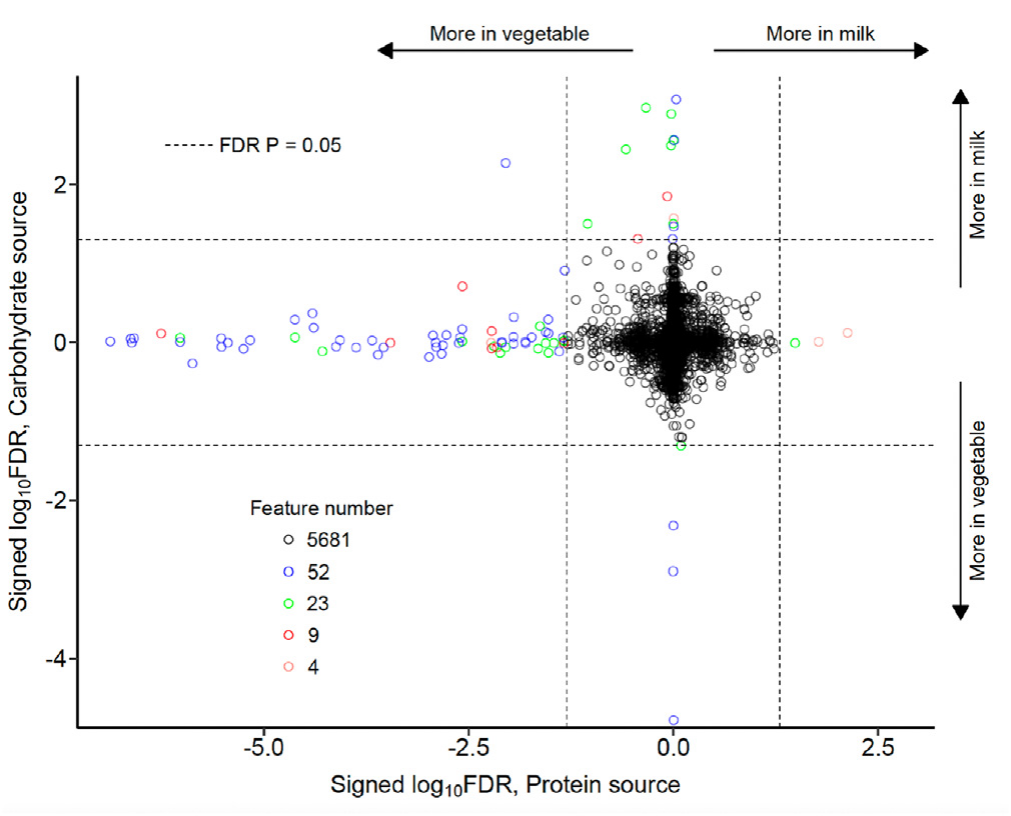
Plot of all metabolomic features comparing children in the milk matters study. Each of the 5769 features are represented by an open circle. The color of the circle indicates the biochemical category of the feature. Along the horizontal orientation, features are plotted by the strength of association with milk protein consumption. Along the vertical orientation, features are plotted by the strength of association with milk carbohydrate (lactose) consumption. The strength of association is expressed as the log_10_ of the FDR. More than 98.5% of all features are not significantly associated with either dietary intake characteristic. Among those 95 features that are associated with consumption of a protein or carbohydrate source, 76% associate with vegetable protein intake, and 17% associate with milk carbohydrate consumption. The plot was composed from data from 200 children, 50 from each of the 4 food groups. FDR, false discovery rate.

Among the 5769 unique fecal metabolic features detected, 88 (1.5%) were found to be associated with consumption of either MP or MC (Figure 4). Among the 21 features associated with dietary carbohydrate source, 16 were associated with MC and 5 with VC. The features associated with MC consumption were pyrimidine nucleosides, whereas aryl ketones and amino acids were associated with VC consumption. Among the 75 features associated with dietary protein source, 3 were associated with MP and 72 with VP. Those features associated with MP consumption were cholestane steroids and ceramides, typically found in dairy products. Many of the 72 features associated with VC consumption were flavonoids, amino acids, and heterocyclic, nitrogen-containing, aromatic ring structures. These features are indicative of common phytochemicals seen in a multitude in plants.

**FIGURE 5:**
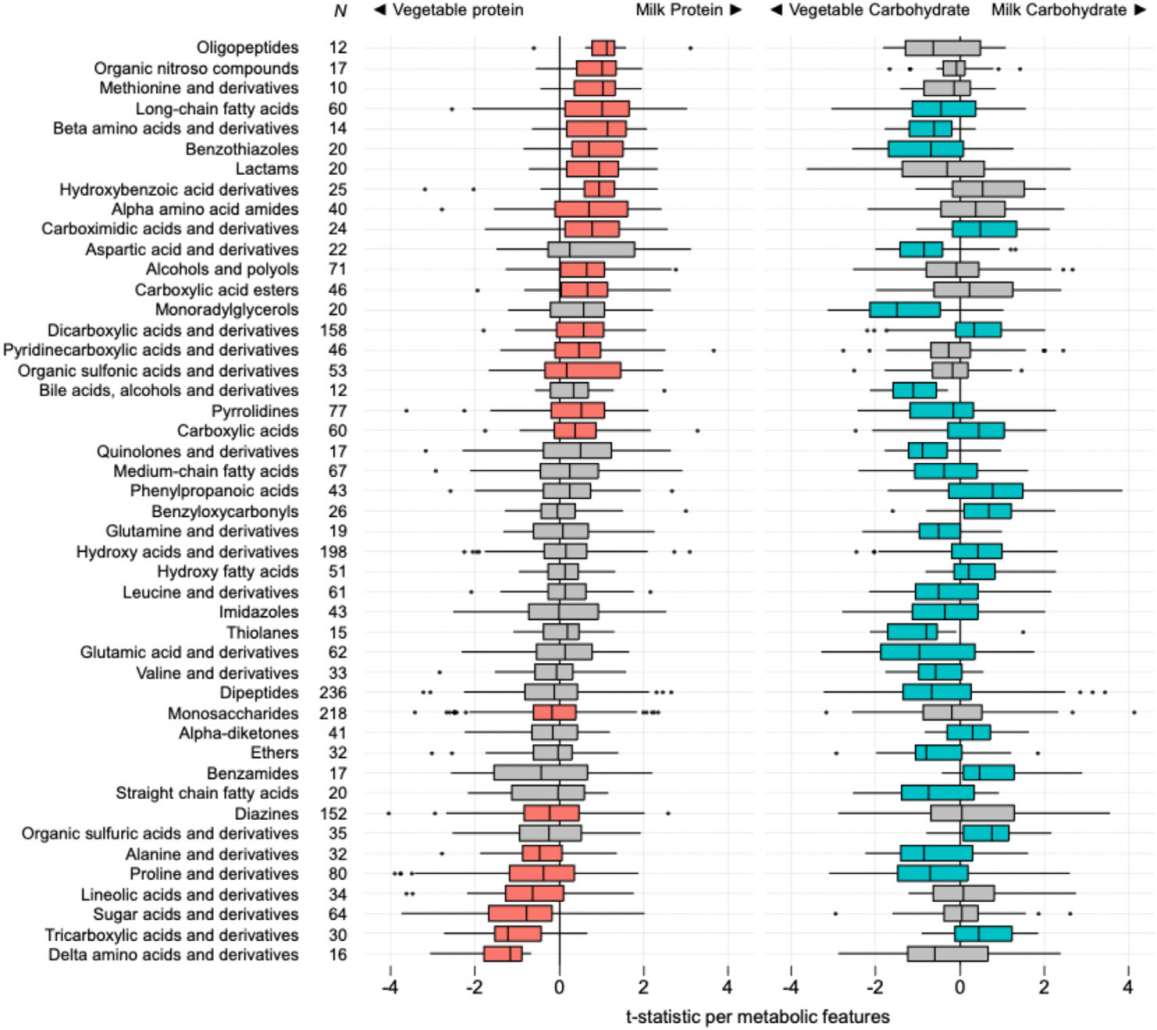
Metabolic enrichments in milk protein and milk carbohydrate consumption. Wilcoxon signed-rank tests (2-tailed) was performed to the individual differential abundance trends of metabolites to identify compound classes enriched in certain milk consumption group; *t* statistics per metabolic features were calculated from generalized linear model. Among the 46 compound classes (except for isoflav-2-enes), 25 and 31 classes were significantly enriched by milk protein and milk carbohydrate consumption [false discovery rate (FDR) < 0.05]; 17 of 25 classes (68.0%) in milk protein consumption and 11 of 31 classes (35.5%) in milk carbohydrate consumption were positively enriched, which means that their member in class tended to be more abundance in group consuming milk protein or milk carbohydrate. Eight of 25 classes (32.0%) in milk protein consumption and 20 of 31 classes (64.5%) in milk carbohydrate consumption were negatively enriched, which means that their member in class tended to be more abundance in control groups consuming vegetable ingredients. Boxplot boxes indicate the first, second, and third quartiles of the data. The boxes filled in color were for significantly enriched classes. Boxplot whiskers indicate the inner fences of the data, with points outside the inner fences plotted as outliers. The plot was composed from data from 200 children, 50 from each of the 4 food groups.

When the metabolic features were categorized by compound class, several classes were seen in greater abundance with MP and milk MC consumption (Figure 5). The classes seen in greater abundance with MP consumption include oligopeptides, methionine, long-chain fatty acids, α and β amino acids, organic sulfonic acids, and carboximidic acids. The classes seen in greater abundance with MC consumption include phenylpropanoic acids, hydroxy acids, hydroxy fatty acids, benzamides, and tricarboxylic acids. The classes such as carboxymidic acids, dicarboxylic acid, and carboxylic acids showed greater abundance with either MP or MC consumption.

When the 3 supplementary foods that contained components of milk (MPMC, VPMC, and MPVC) were compared with VPVC, we found the following among fecal metabolomic features (Supplemental Tables 4–6): *1*) VP consumption increased the abundance of phytochemicals; *2*) lactose consumption increased the abundance of pyrimidines, dipeptides, nucleosides, and monosaccharides; and *3*) MP with lactose consumption increased the abundance of measured amino acids. In contrast, children who consumed food containing MPVC had lower fecal concentrations of fecal amino acids than children who consumed VPVC or VPMC.

**FIGURE 6:**
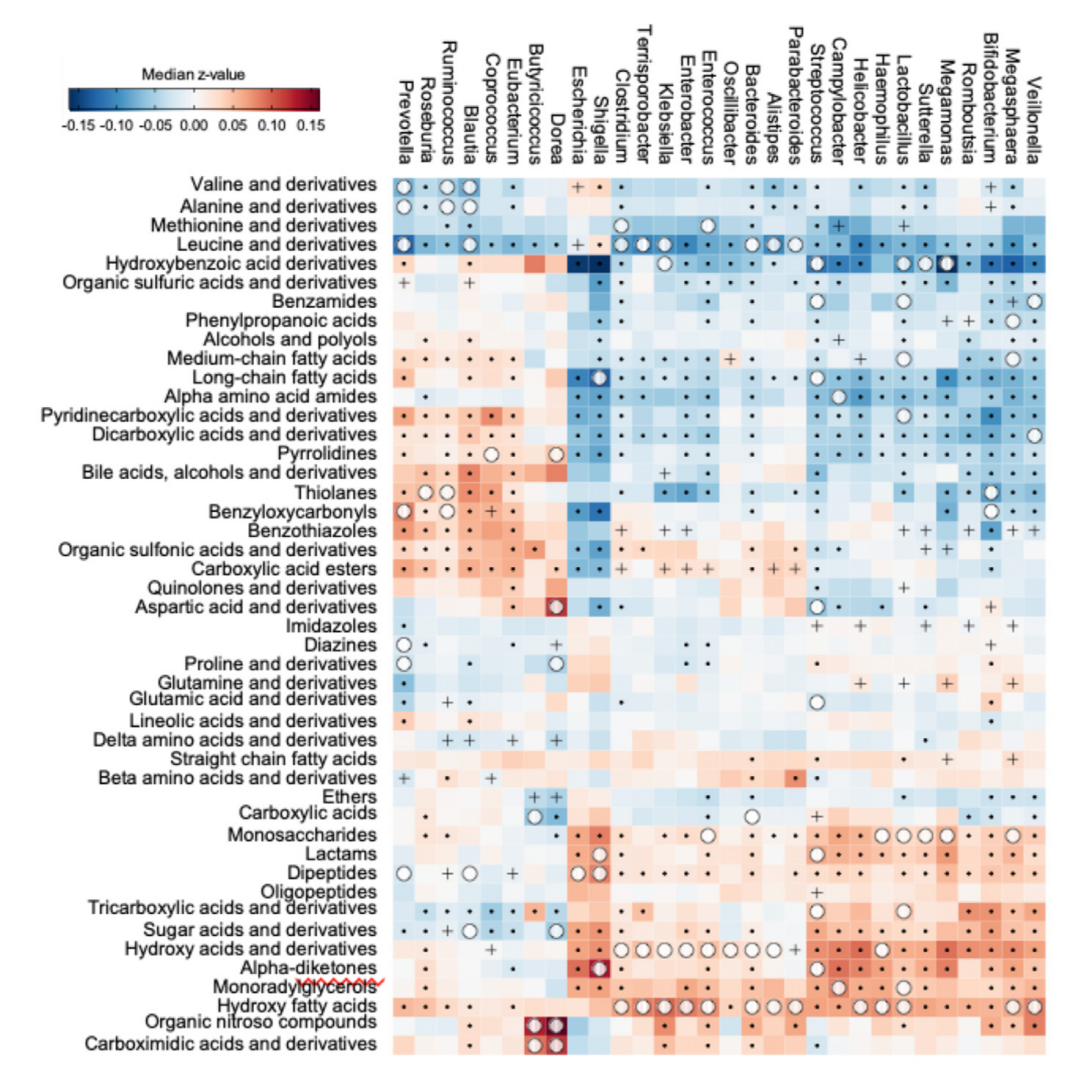
Correlations between fecal microbial genus and compound classes in moderate malnutrition. Correlations were linked by false discovery rate (FDR)-significant, Spearman rank correlation between microbial taxa and metabolites. Among the 629,393 linkages between 257 microbial taxa and 2449 metabolic features, we found 6444 significant linkages (FDR < 0.05). To test the enrichment of correlation, Spearman ρ were transformed to Fisher *z* values for normal distribution, Wilcoxon signed-rank tests (2-tailed) was performed to the individual differential abundance trends of covariation in each linking between genus and classes. In addition, Fisher exact test was performed using 6444 significant linkages between taxa and metabolites (FDR < 0.05) to test whether connectivity between genus and compound class is significant among genus and compound classes detected in correlation test. (Significant when odd ratio > 1 and *P* value < 0.05.) The black dots depict significant correlations between genus and compound class determined by a Wilcoxon signed-rank test; black cross depicts significant correlation determined by Fisher exact test; and white circle depicts significant correlation in both tests. The coloring of heatmap represents median Fisher *z* value of taxa-metabolite linkages in correlation between genus and class. The plot was composed from data from 200 children, 50 from each of the 4 food groups.

Multiple fecal genus taxa were associated with fecal metabolite compound class abundance (Figure 6). The nitroso compounds and carboximidic acids, including organonitrogen residue, were positively associated with *Dorea* and *Butyricicoccus* species. The methionines, α-amino acids, and long-chain fatty acids abundant in MP consumption were negatively associated with *Clostridium*, *Enterococcus*, *Streptococcus*, and *Shigella* species. The carboxylic acids and dicarboxylic acids, abundant in both milk consumption groups, were negatively associated with *Dorea*, *Bacteroides*, and *Veillonella* species. Among the classes greater in MC consumption, hydroxy acids and hydroxy fatty acids had positive correlation with multiple genus greater than other classes. Tricarboxylic acids were positively associated with *Streptococcus* and *Lactobacillus* species, whereas benzamides were negatively associated with *Streptococcus*, *Lactobacillus*, and *Veillonella* species. Phenylpropanoic acids were negatively associated with *Megasphaera* species.

The rate of weight gain among children consuming MPMC was greater than that of children consuming VPVC (difference: 0.3 g/kg/d; 95% CI: 0.0, 0.6; *P* = 0.03) (Supplemental Table 7). Clinical outcomes were otherwise similar, including recovery. Compared with VPVC, MPMC had a similar rate of recovery (RR: 1.08; 95% CI: 0.94, 1.24; *P* = 0.28), as did MPVC (RR: 0.99; 95% CI: 0.86, 1.15; *P* = 0.92) and VPMC (RR: 1.03; 95% CI: 0.90, 1.19; *P* = 0.67).

## Discussion

This clinical trial demonstrated that intestinal permeability as measured by lactulose permeability was unaffected by supplementary feeding for 4 wk with foods that contain MP, MC, or both, as compared with a food containing no milk ingredients. Irrespective of food group, there was no change in %L after 4 weeks of feeding. The fecal 16S rRNA gene configuration showed no differences whether children with MAM received supplementary feeding with or without MP, MC, or both for 1 wk, as evidenced by similar β-diversity, relative taxa abundances, and α-diversity. These fecal findings are unexpected, because one of the primary determinants of the fecal 16S rRNA gene configuration is widely thought to be the diet [22]. The untargeted fecal metabolomic analyses show that MP consumption increases amino acid and fatty acid metabolite abundance. MC consumption increases nucleoside and short-chain fatty acid abundance. The untargeted fecal metabolomics also found differences that are consistent with the consumption of the assigned supplementary foods, supporting participant adherence.

A limitation of the study is that the findings from this population with its habitual diet of rice porridge may not apply to other healthy or malnourished populations. A further limitation is that not all participants who met criteria for providing primary outcome data could be analyzed, in large part owing to defaulting or clinical deterioration to SAM before sample collection visit. Despite these limitations, the strong study design with randomization by child and blinding of the participants and study personnel minimizes the possibility that the findings were biased by design. The relatively small number of untargeted metabolomic samples analyzed from each dietary group, 50, were inadequate to determine differences in feature abundance <20%. Very strict criteria for the FDR, *P* < 0.05, may have obscured some important metabolomic differences. Nevertheless, the analytical methods used to interrogate the feces were robust and have been used in multiple settings previously. The stark differences in the nutrient composition of the supplementary foods, and unavailability of milk in the habitual diet, suggest these findings are not tainted by undocumented milk consumption.

The persistent increased %L indicates that damaged upper small bowel requires >4 wk to heal. Although the half-life of the gut epithelium is 11 days, multiple complete regenerations of the gut epithelium are likely needed to restore normal function. Nearly two-thirds of children recovered by anthropometry, suggesting children with MAM are able to absorb adequate nutrients to support lean mass accretion despite persistent barrier dysfunction. Our findings are consistent with observations in Malawian children with kwashiorkor, who had no demonstrable change in their dual sugar permeability tests after 4 wk of therapy, despite substantial clinical improvement [23]. This observation raises the following questions: “Can gut integrity be restored more quickly with the use of gut-directed pharmacologic therapy?” and “Might better long term gut health be achieved with a longer nutritional intervention?”

The fecal 16S rRNA gene configuration finding in these children with MAM is consistent with a previous study in which cohorts of children with SAM were fed a therapeutic food with or without 18% weight-by-weight oats [7]. Dietary oats did not result in any differences in the fecal 16S rRNA gene configuration. Animal studies indicate that rapid alterations occur in the 16S rRNA gene configuration of the ileum after the consumption of various diets within a matter of hours [24]. In humans, there are substantial differences between ileal and colonic microbial populations [[25], [26], [27]].

The initial 16S rRNA gene sequencing conducted in this study yielded ∼35,000 reads per sample. In the subset of children whose fecal samples were sequenced to a median of 22.5-fold greater depth, 16S rRNA gene sequencing revealed no substantial differences in numbers of species identified, their relative abundances, or in diversity comparisons. Thus, the similarity of the 16S rRNA gene findings among children consuming remarkably different diets is unlikely to be an artifact of the 16S rRNA gene sequencing depth. These findings suggest that 16S rRNA gene sequencing of fecal samples may represent simply the taxa present in the distal colon. 16S rRNA gene results should not be presumed to represent the taxa in the other regions of the gastrointestinal tract, such as the ileum. Unfortunately, the scientific literature includes a multitude of reports in which fecal 16S rRNA gene configurations were determined, and these data were interpreted as indicative of the entire human gut [[28], [29], [30], [31]]. The observations of this study suggest this inference may be speculative.

More sensitive methods to detect diet-induced differences of intestinal microbial configurations, such as untargeted, deep metagenomic sequencing and digital drop PCR, may elucidate such differences. A recent cross-sectional study focused on campylobacter ecology in asymptomatic Ethiopian children interrogated the same stool specimen using 3 different methods [32]. Among 100 children, 9% were positive for campylobacter using 16S rRNA gene multiamplicon sequencing, 50% were positive using custom targeted PCR probes, and 88% were positive using meta-total RNA sequencing processed with the IDseq pipeline at a depth of 4 million reads per specimen. The determination of taxa abundance differs with sensitivity of the analytical and bioinformatic methods used, and the clinical importance of these of these differences is uncertain.

Analogously, our group has demonstrated that host mRNA originating from the small bowel can be reliably detected in feces with digital drop PCR, when very low copy numbers are present (50–500 copies) [33]. The untargeted metabolomic analyses suggest that consumption of MP or MC increases the luminal availability of a small number of nutrients in malnourished children. Because this increase is seen with MC consumption without MP, this may reflect bacterial metabolism of lactose in the ileum and colon [34]. Overall, the untargeted fecal metabolomic composition was similar between the 4 dietary groups, with the differences identified in <2% of the metabolites. There were strong associations seen between fecal bacterial taxa and fecal metabolite compound class. These findings support the notion that the composition of the feces is largely unaffected by substantial changes in the diet and that fecal metabolites by-in-large originate from the fecal microbes.

The study findings did not determine whether MP or MC was responsible for improvements in gut permeability or changes in the microbial or the metabolomic milieux. Gut permeability was not improved over 4 wk of feeding, and the fecal analyses undertaken did not demonstrate consistent patterns of fecal microbiota or metabolomic change. Future studies may explore the duration of increased intestinal permeability after MAM treatment and its response to dietary interventions to guide interventions to prevent persistence and recurrence of MAM among children in resource poor settings.

## Author contributions

The authors’ responsibilities are as follows – DTH, ASK, MJM: designed the study; DTH, KBS, TM, NN, ASK, ASN, MJM: collected the data; ML: supervised and analyzed the 16S rRNA gene data; MS, JHS, YAG: conducted the metabolomic analyses and analyzed and interpreted the mass spectrometry data; MS, TM, KBS: wrote the first draft of the manuscript; MJM: had overall responsibility for the study; and all authors: read, edited, and accepted the final version of the manuscript.

## Funding

The project was funded by the Danish Dairy Research Foundation. The funding source had no role in the design or conduct of the study, data collection or analyses, nor in the interpretation of the findings. The funder did not approve the manuscript before submission nor was it consulted about the decision to submit the manuscript for publication.

## Data availability

This is an open access article. Deidentified individual participant data that underlie the reported results will be made available 3 mo after publication at WashU Research Data (data.library.wustl.edu). The study protocol is available online as a supplement to this publication.

## Conflict of interest

MJM received funding from the Danish Dairy Research Foundation for this work and is an Associate Editor at the *American Journal of Clinical Nutrition*. All other authors report no conflicts of interest.
