# Supplementary material for: The Impact of Milk on Gut Permeability, Fecal 16S rRNA Gene Microbiota Profiling, and Fecal Metabolomics in Children with Moderate Malnutrition in Sierra Leone: A Double-Blind, Randomized Controlled Trial
Source: Am J Clin Nutr. 2024 Sep 21;120(5):1114–24. doi: 10.1016/j.ajcnut.2024.09.018 (PMC11600093; doi:10.1016/j.ajcnut.2024.09.018)
Supplement: multimedia component 1 [file mmc1.pdf]

Supplementary Materials

Supplementary Methods Metabolomic isolation and detection methods
Supplementary Table 1 Lactulose excretion at baseline and after 4 weeks
Supplementary Table 2 Change in α diversity from enrollment to week 4
Supplementary Table 3 Regression modeling of determinants of α diversity week 4
Supplementary Table 4 Metabolomic features associated with milk or vegetable protein consumption
Supplementary Table 5 Metabolomic features associated with milk or vegetable carbohydrate consumption
Supplementary Table 6 Metabolomic features associated with milk consumption
Supplementary Table 7 Clinical outcomes of full cohort
Supplementary Figure 1 Enrollment, randomization, and outcomes of study participants
Supplementary Figure 2 Change in lactulose excretion from baseline to 4 weeks
Supplementary Figure 3 α diversity week 4
Supplementary Figure 4 Change in α diversity from enrollment to week 4
Supplementary Figure 5 Twenty most abundant taxa identified by deep 16S rRNA sequencing
Supplementary Figure 6 α and β diversity week 4 calculated using only 16S rRNA sequencing.

This supplemental material has been provided by the authors to give readers additional information about their work.

Supplementary Methods

Profiling Metabolic Features from Fecal Samples

Extracted samples, aqueous and organic extractions, were dried and reconstituted with 50% methanol and sonicated and vortexed vigorously then centrifuged briefly. The supernatant fraction was transferred to the mass spectrometry auto-sampler vial and injected into mass spectrometry. The aqueous extraction was run using the three methods of liquid chromatography tandem mass spectrometry (LC-MS/MS); C18 column in positive mode for middle polar features, and amino acids (C18-Positive), HILIC column in positive mode for positive polar features, amines and aromatic metabolites (HILIC-Positive), and Amide column in negative mode for negative polar features, carbohydrates and acidic metabolites (Amide-Negative). The organic extraction was run on C8 column in positive mode to measure hydrophobic features and lipids (C8-Positive). The detailed LC-MS methods are as follows:

(i) For C18-Positive method, 5 µL of aqueous extraction was injected on the ACQUITY UPLC HSS T3 column (2.1 x 150mm, 1.7 µm; Waters). The column was isocratically equilibrated at a flowrate of 0.4 mL/min, 30 °C oven temperature with 98% of mobile phase A (0.1% Formic Acid in Water) for 2 min, followed by a linear gradient to 27% of mobile phase B (0.1% Formic Acid in 90% Acetonitrile) over 7 min, a linear gradient to 98% of mobile phase B over 7 min, and then 2 min at 98% of mobile phase B. The MS1 full scan was performed over 100–600 m/z at 60,000 resolution, using heated electrospray ionization in the positive ion mode; (ii) for HILIC-Positive method, 5 µL of aqueous extraction was injected on the Atlantis Premier BEH zwitterionic-HILIC (2.1 x 150mm, 1.7 µm; Waters). The column was isocratically equilibrated at a flowrate of 0.5 mL/min, 32 °C oven temperature with 95% of mobile phase B (0.1% Formic Acid in Acetonitrile) for 3 min followed by a linear gradient to 80% of mobile phase B over 7 min, a linear gradient to 50% of mobile phase A (10 mM Ammonium Acetate with 0.1% Acetic Acid in 5% Methanol) over 5 min, and then 4 min at 50% of mobile phase A. The MS1 full scan was performed over 70–800 m/z at 60,000 resolution, using heated electrospray ionization in the positive ion mode; (iii) for Amide-Negative method, 5 µL of aqueous extraction was injected on the BEH Amide column (2.1 x 150mm, 1.7 µm; Waters). The column was isocratically equilibrated at a flowrate of 0.5 mL/min, 50 °C oven temperature with 95% of mobile phase B (Acetonitrile, pH 9) for 3 min followed by a linear gradient to 80% of mobile phase B over 7 min, a linear gradient to 50% of mobile phase A (20 mM Ammonium Acetate in Water, pH 9) over 5 min, and then 4 min at 50% of mobile phase A. The MS1 full scan was performed over 70–800 m/z at 60,000 resolution, using heated electrospray ionization in the negative ion mode; and (iv) for C8-Positive method, 5 µL of

organic extraction was injected on the ACQUITY UPLC BEH C8 column (2.1 x 100mm, 1.7 µm; Waters). The column was isocratically equilibrated at a flowrate of 0.4 mL/min, 30 °C oven temperature with 80% of mobile phase A (10 mM Ammonium Acetate with 0.1% Acetic Acid in 5% Methanol) for 1 min, followed by a linear gradient to 80% of mobile phase B (0.1% Acetic Acid in Methanol) over 2 min, a linear gradient to 100% of mobile phase B over 7 min, and then 3 min at 100% of mobile phase B. The MS1 full scan was performed over 50–1100 m/z at 60,000 resolution, using heated electrospray ionization in the positive ion mode.

The 'Deep Scan' mode of AcquireX MS2 data acquisition was applied for every batch using the samples pooled by batches with generating 1 inclusion lists followed by 3 times of exclusion override (Inclusion list peak fragmentation threshold, 50%; Exclusion duration, 10 sec). Additional MS settings are as follows: the capillary voltage in the positive mode 3.4 kV, negative mode 2.4 kV, ion transfer tube temperature 325 °C, vaporizer temperature was 350 °C, normalized collision energy 35%.

Quality Control of Fecal Metabolite Profiling

For a comprehensive profiling, extractions of 200 individual samples were randomly allocated into 10 analytical batches. Random allocation was repeated 5,000 times using R to distribute the samples equally according to the following 6 variables: food groups, age, sex, breastfeeding, weight gain over 2.5 g/kg/day in 4 weeks, and outcome. The case showing the lowest average P values of the six variables by Chi-square test was selected for data acquisition sequence. For monitoring the instrument and column performance, QrESS standard mixture (Cambridge Isotope Laboratories, Inc.) and Total QC, a pooling of entire samples, were run at starting point and between every batch.

Metabolomics Data Post-processing and Analysis

We used Compound Discoverer 3.3 (Thermo Fisher Scientific) to process raw LC-MS data for spectra selection, retention time alignment between samples, grouping all the detected features by molecular weight and retention time, calculating area under curve feature abundances, and assigning putative class and/or metabolite annotation by elemental composition prediction and spectral library matching. The following parameters were used for the feature detection and grouping: mass tolerance 5 ppm, minimum peak intensity 1e4, retention time tolerance 0.2 min, signal-to-noise threshold 3, peak rating threshold 5 in at least 10% of samples. The gaps for missing peaks were filled with re-detected peak, matching ion, spectrum noise, and trace area. The value was considered as full gap if the gap was filled with spectrum noise or trace

area, and the features without full gap at least in 20% of samples were used for further processing. The area under curve data was normalized for batch effect correction by locally estimated scatterplot smoothing signal correction (QC-RLSC) using Total QCs. The parameters for QC-RLSC normalization are as follows: minimum QC coverage 30%, and maximum QC area relative standard deviation 10%. The QC-RLSC normalized data were centered for adjusting injection amount difference between samples. This comprehensive feature list contains a continuous, numerical value between -1 and 1 for the signal strength for every clinical sample analyzed, a measure of the quantity of that feature. The features lower than 2 of sample to blank ratio were marked as background and excluded for further analysis. The parameters for predicting elemental compositions are as follows: mass tolerance 5 ppm, maximum ring-and-double-bonds-equivalent 40, minimum Hydrogen to Carbon ratio 0.1, maximum Hydrogen to Carbon ratio 3.5, intensity tolerance 30%, signal-to-noise threshold 3, minimum spectral fit 30%, and minimum pattern coverage 90%. The 5,775 features characterized by the above workflow were defined as metabolite features and further annotated by putative compound class and metabolic identities. For the library search, the databases from mzCloud, MassBank of North America (MoNA) [http://mona.fiehnlab.ucdavis.edu], Global Natural Product Social Molecular Networking (GNPS) [https://www.nature.com/articles/nbt.3597], National Institute of Standards and Technology (NIST20), and LipidBlast [https://www.nature.com/articles/nmeth.2551]. The following parameters were used for library matching: precursor and fragment ion mass tolerance 10 ppm, minimum matching score 0.5 (Cosine algorithm for mzCloud and HighChem HighRes algorithm for other databases), and ion activation energy tolerance 20. Any library matches were considered as level two according to the metabolomics standards consortium guidelines [https://link.springer.com/article/10.1007/s11306-007-0082-2]. The putative compound class was predicted by matching with Human Metabolome Database (HMDB) [https://doi.org/10.1093/nar/gkab1062] using accurate mass of precursor ion and predicted elemental composition and major class among results was assigned.

Supplementary Table 1 Lactulose excretion at baseline and after 4 weeks

Lactulose excretion results at baseline and after 4 weeks of treatment among Sierra Leonean children with higher risk moderate wasting (MUAC < 12.0 cm) who received supplementary foods with different milk and vegetable protein and carbohydrate content[1]

[1] Values are presented as median (IQR), unless otherwise indicated. MPMC, milk protein milk carbohydrate; MPVC, milk protein vegetable carbohydrate; VPMC,

	Milk protein Milk carbohydrate (MPMC)	Milk protein Vegetable carbohydrate (MPVC)	Vegetable protein Milk carbohydrate (VPMC)	Vegetable protein Vegetable carbohydrate (VPVC)	MPMC vs. VPVC *P* value[2]	MPVC vs. VPVC *P* value[2]	VPMC vs. VPVC *P* value[2]
Baseline	N = 46	N = 53	N = 46	N = 56			
Lactulose, %	0.22 (0.15, 0.37)	0.28 (0.16, 0.48)	0.21 (0.13, 0.34)	0.31 (0.14, 0.39)	0.30	0.94	0.15
Week 4[3]	N = 97	N = 97	N = 105	N = 94			
Lactulose, %	0.28 (0.16, 0.50)	0.30 (0.13, 0.34)	0.28 (0.17, 0.46)	0.27 (0.15, 0.46)	0. 66	0.11	0.70
Lactulose ≥ 0.2, n (%)	63 (65)	71 (73)	72 (69)	62 (66)	0.59	0.54	0.75
Change, median of differences (95% CI)[4]	N = 26	N = 34	N = 34	N = 37			
Lactulose, %	0.05 (-0.02, 0.14)	0.02 (-0.12, 0.13)	0.05 (-0.04, 0.13)	-0.02 (-0.12, 0.09)	0.93	0.22	0.85

vegetable protein milk carbohydrate; VPVC, vegetable protein vegetable carbohydrate.

[2] For baseline and week 4 comparisons, *P* values were calculated using the Wilcoxon rank-sum test. For the change from baseline to week 4 comparisons, *P* values were calculated using ordinal logistic regression, with change in %L excretion (week 4 – baseline) as the dependent variable, study group as the independent variable, and baseline lactulose excretion as a covariate.

[3] Two measurements were taken after four weeks of supplementary feeding (milk protein / milk carbohydrate n = 1, milk protein / vegetable carbohydrate n = 1).

[4] Change in %L excretion was calculated by subtracting the baseline value from the week 4 value. The Hodges-Lehmann estimator was used to generate the median of the differences between groups as well as 95% CIs.

Supplementary Table 2 Change in α diversity from enrollment to week 4

Fecal 16S microbiome α-diversity indices at baseline and after 4 weeks of treatment among Sierra Leonean children with higher risk moderate wasting (MUAC < 12.0 cm) who received supplementary foods with different milk and vegetable protein and carbohydrate content[1]

Alpha diversity measure	Milk protein Milk carbohydrate (MPMC)	Milk protein Vegetable carbohydrate (MPVC)	Vegetable protein Milk carbohydrate (VPMC)	Vegetable protein Vegetable carbohydrate (VPVC)	MPMC vs. VPVC *P* value[2]	MPVC vs. VPVC *P* value[2]	VPMC vs. VPVC *P* value[2]
Baseline	N = 32	N = 27	N = 29	N = 34			
Shannon	4.37 (4.08, 4.60)	4.02 (3.90, 4.66)	4.39 (3.64, 4.72)	4.24 (3.66, 4.45)	0.25	0.59	0.28
Chao1	57.4 (45.5, 68.8)	54.0 (43.3, 67.7)	60.2 (52.5, 65.2)	51.7 (35.0, 64.2)	0.35	0.54	0.12
Week 4[3]	N = 95	N = 96	N = 95	N = 88			
Shannon	4.30 (3.85, 4.71)	4.21 (3.82, 4.60)	4.25 (3.74, 4.55)	4.20 (3.81, 4.55)	0.23	0.79	0.66
Chao1	55.6 (45.2, 67.0)	55.6 (45.2, 67.0)	55.6 (45.2, 67.0)	55.6 (45.2, 67.0)	0.28	0.75	0.84
Change, median of differences (95% CI)[4]	N = 24	N = 23	N = 20	N = 26			
Shannon	-0.02 (-0.30, 0.27)	0.20 (-0.05, 0.48)	-0.17 (-0.52, 0.22)	-0.06 (-0.21, 0.48)	0.28	0.67	0.22
Chao1	-2.2 (-9.1, 4.7)	3.9 (-3.2, 13.2)	-8.3 (-14.6, 1.5)	3.0 (-6.5, 14.2)	0.59	0.13	0.15

[1] Values are presented as median (IQR), unless otherwise indicated. MPMC, milk protein milk carbohydrate; MPVC, milk protein vegetable carbohydrate; VPMC, vegetable protein milk carbohydrate; VPVC, vegetable protein vegetable carbohydrate.
[2] For baseline and week 4 pairwise comparisons, *P* values were computed using the Wilcoxon rank-sum test. For pairwise comparisons of change in α-diversity indices, *P* values were computed using ordinal logistic regression, with change in α-diversity value (week 4 – week 0) as the dependent variable, study group as the independent variable, and baseline measure as a covariate.
[3] Several samples were collected later than 4 weeks due to no-shows or inability of child to pass stool sample on day of study (milk protein milk carbohydrate n = 5, milk protein vegetable carbohydrate n = 6, vegetable protein milk carbohydrate n = 8, vegetable protein vegetable carbohydrate n = 5).
[4] Change in diversity index was calculated by subtracting the baseline value from the week 4 value. The Hodges-Lehmann estimator was used to generate the median of the differences between groups as well as 95% CIs.

Supplementary Table 3

The degree to which variability in Shannon Diversity may be explained by collected demographic, clinical, and diet variables was assessed using linear regression, after confirming residual normality and homogeneity of variance. Variables considered *a priori* as potentially predictive of Shannon diversity were included in a regression analysis to assess total variance explained in the form of unadjusted and adjusted R^2. The contribution to variance explained of individual variables in the context of the rest of the model was assessed via evaluation of R^2 following serial removal and addition of each variable back into the regression. Clinic location and month of enrollment were modeled as categorical variables. To determine the contribution of month of enrollment as a whole to the model, all variables for month of enrollment were removed and then re-added *en bloc*. The same process was done for clinic, which had multiple categories.

For the entire model, the unadjusted R^2 was 24%, while adjusted R^2 was 10%. The partial R^2 for month of enrollment was 10%, while it was 7% for clinic location and 3.6% for age. Within the context of the model, presence of milk protein contributed a partial R^2 of 0.2%, while milk carbohydrate contributed a partial R^2 of 0.01%. When modeled without additional covariates, milk protein (R^2 = 0.02%) and milk carbohydrate (R2 = 0.001%) explained little variance in Shannon Diversity. The majority of variability in Shannon Diversity was unexplained by the available variables.

The variables of the model and their contribution to R^2 are shown in tabular form below.

Model term	Estimate	95% CI	P value	Partial R^2
Age, months	0.026	(0.01, 0.05)	0.011	0.036
Animals in home	0.039	(-0.23, 0.3)	0.769	0.000
Breastfeeding status	0.001	(-0.29, 0.29)	0.994	0.000
Clinic at Bendu	-0.039	(-0.45, 0.37)	0.853	0.000
Clinic at Bumpeh	-0.100	(-0.52, 0.32)	0.642	0.000
Clinic at Futa	-0.160	(-0.62, 0.3)	0.495	0.000
Clinic at Gbongay	-0.396	(-0.95, 0.16)	0.163	0.000
Clinic at Gobaru	-0.347	(-0.86, 0.17)	0.184	0.000
Clinic at Hongai	-0.426	(-0.82, -0.03)	0.033	0.000
Clinic at Konia	-0.364	(-1.17, 0.45)	0.376	0.000
Clinic at Makoma	-0.385	(-1.21, 0.44)	0.358	0.000
Clinic at Malema	-0.294	(-0.7, 0.11)	0.153	0.000
Clinic at Potoru	0.013	(-0.43, 0.46)	0.954	0.000
Clinic at Taninahun	-0.580	(-1.24, 0.08)	0.084	0.000
Clinic at Zimmi	0.238	(-0.28, 0.76)	0.368	0.000
Enrolled in December	-0.549	(-1.49, 0.39)	0.251	0.000
Enrolled in February	-0.581	(-1.5, 0.34)	0.214	0.000
Enrolled in January	-0.595	(-1.46, 0.27)	0.174	0.000
Enrolled in June	-1.089	(-1.94, -0.23)	0.013	0.000
Enrolled in March	-0.024	(-1.14, 1.09)	0.966	0.000
Enrolled in May	-0.834	(-1.69, 0.03)	0.057	0.000
Enrolled in November	-0.730	(-1.76, 0.3)	0.163	0.000
Milk protein	0.055	(-0.14, 0.25)	0.574	0.002
Milk carbohydrate	-0.018	(-0.2, 0.17)	0.852	0.000
Sex	0.067	(-0.13, 0.27)	0.513	0.002
Stunted at baseline	-0.039	(-0.26, 0.18)	0.732	0.001

Supplementary Table 4

Fecal metabolomic features increased or decreased by milk protein consumption

Compound ID	Putative Class (Annotation)	Abundance in MPVC	Abundance in VPVC	*P* comparison	False Discovery Rate, Q value
Hilic-pos-000661	Amines	-0.171 ± 0.010	0.010 ± 0.024	2.71E-11	1.57E-07
Amide-neg-000980	Isoflav-2-enes (Daidzein)	0.001 ± 0.009	0.129 ± 0.017	3.57E-09	1.03E-05
C18-pos-000858	Amines	-0.018 ± 0.008	0.102 ± 0.018	8.75E-09	1.66E-05
C18-pos-000537	Amines	0.050 ± 0.006	0.176 ± 0.020	1.15E-08	1.66E-05
Amide-neg-000144	Flavones (Daidzein-4'-SO_4)	-0.020 ± 0.008	0.102 ± 0.019	9.96E-08	1.15E-04
Amide-neg-001204	Isoflav-2-enes (Rubiadin)	-0.081 ± 0.004	-0.003 ± 0.014	1.37E-06	1.23E-03
C18-pos-000980	Amines	-0.033 ± 0.006	0.046 ± 0.014	1.49E-06	1.23E-03
Amide-neg-001696	Isoflav-2-enes	-0.018 ± 0.004	0.050 ± 0.012	3.37E-06	2.43E-03
C18-pos-007987	α amino acids	-0.131 ± 0.006	-0.064 ± 0.013	5.80E-06	3.72E-03
C18-pos-000269	Isoflav-2-enes (Daidzein)	-0.142 ± 0.007	-0.039 ± 0.022	8.90E-06	4.93E-03
Amide-neg-011129	α amino acids	-0.248 ± 0.005	-0.186 ± 0.012	9.39E-06	4.93E-03
C18-pos-015576	N-acyl-α amino acids	-0.098 ± 0.006	-0.045 ± 0.009	3.00E-05	1.37E-02
Amide-neg-000894	Isoflav-2-enes (Genistein)	-0.006 ± 0.012	0.094 ± 0.019	3.09E-05	1.37E-02
Amide-neg-004969	Isoflav-2-enes	-0.197 ± 0.005	-0.136 ± 0.014	4.31E-05	1.77E-02
Amide-neg-002830	Isoflav-2-enes	-0.072 ± 0.005	-0.010 ± 0.015	4.59E-05	1.77E-02
C18-pos-022512	Phenol ethers	-0.136 ± 0.004	-0.090 ± 0.009	7.34E-05	2.52E-02
C18-pos-003645	Flavones	-0.086 ± 0.008	0.000 ± 0.019	7.41E-05	2.52E-02
C18-pos-001496	Isoflav-2-enes	-0.102 ± 0.009	-0.015 ± 0.021	9.79E-05	3.14E-02
C18-pos-002549	Toluenes	-0.051 ± 0.009	0.011 ± 0.013	1.20E-04	3.64E-02
C18-pos-007435	Phenylpropanes	-0.028 ± 0.013	0.050 ± 0.016	1.28E-04	3.71E-02
C8-pos-003522	Steroid lactones	0.070 ± 0.008	0.130 ± 0.014	1.49E-04	4.01E-02
Hilic-pos-011798	α amino acids	-0.094 ± 0.005	-0.051 ± 0.009	1.53E-04	4.01E-02
C18-pos-004391	Stilbenes	-0.100 ± 0.004	-0.041 ± 0.013	1.84E-04	4.62E-02

Note all of these features were seen in greater abundance among vegetable protein consumers

Supplementary Table 5

Fecal metabolomic features increased or decreased by milk carbohydrate (lactose) consumption

Compound ID	Putative Class (Annotation)	Abundance in VPMC	Abundance in VPVC	*P* comparison	False Discovery Rate, Q value
C18-pos-012811**	Imidazopyrimidines	-0.084 ± 0.013	-0.156 ± 0.004	1.60E-10	9.27E-07
C18-pos-000677^^	Aryl ketones	0.020 ± 0.014	0.168 ± 0.018	1.60E-08	4.63E-05
Hilic-pos-000678**	Pyrimidine nucleosides	0.031 ± 0.014	-0.048 ± 0.007	3.09E-08	5.95E-05
Hilic-pos-000574**	Pyrimidine nucleosides	0.051 ± 0.014	-0.020 ± 0.007	1.77E-07	2.56E-04
C18-pos-010376**	Imidazopyrimidines (fenethylline)	-0.062 ± 0.013	-0.126 ± 0.007	6.19E-07	7.15E-04
C18-pos-042082**	Benzoxazines	-0.050 ± 0.007	-0.086 ± 0.003	2.21E-06	2.12E-03
Hilic-pos-001455**	Pyrimidine nucleosides	0.099 ± 0.016	0.028 ± 0.007	3.30E-06	2.72E-03
Hilic-pos-003181**	Pyrimidine nucleosides	0.092 ± 0.010	0.030 ± 0.007	6.64E-06	4.80E-03
Hilic-pos-000534**	Pyrimidine nucleosides	0.022 ± 0.016	-0.043 ± 0.007	9.97E-06	6.40E-03
Hilic-pos-008275**	Azacyclic compounds	0.008 ± 0.013	-0.048 ± 0.007	1.53E-05	8.48E-03
Hilic-pos-000994**	Pyrimidine nucleosides	0.128 ± 0.012	0.075 ± 0.007	1.61E-05	8.48E-03
Amide-neg-004553**	Dipeptides (Glutamate/ glycine)	-0.034 ± 0.013	-0.093 ± 0.006	2.15E-05	1.03E-02
Hilic-pos-001272**	Monosaccharides	0.134 ± 0.014	0.062 ± 0.012	3.90E-05	1.73E-02
C18-pos-002347^^	Quinoline carboxylic acids	-0.090 ± 0.011	-0.003 ± 0.017	5.21E-05	2.15E-02
C18-pos-009224**	Azolines	0.090 ± 0.009	0.045 ± 0.005	8.48E-05	3.27E-02

The features designated by ** were found in greater abundance in children consuming milk carbohydrate.
The features designated by ^^ were found in greater abundance in children consuming vegetable carbohydrate.

Supplementary Table 6

Fecal metabolomic features enhanced or diminished by milk protein and milk carbohydrate consumption

Compound ID	Putative Class (Annotation)	Abundance in MPMC	Abundance in VPVC	*P* comparison	False Discover Rate, Q value
Hilic-pos-000661	Amines	-0.184 ± 0.010	0.010 ± 0.024	1.41E-12	8.16E-09
Amide-neg-000980	Isoflav-2-enes (daidzein)	0.004 ± 0.009	0.129 ± 0.017	8.36E-09	1.94E-05
C18-pos-000537	Amines	0.049 ± 0.007	0.176 ± 0.020	1.01E-08	1.94E-05
C18-pos-000677	Aryl ketones	0.019 ± 0.016	0.168 ± 0.018	1.45E-08	2.10E-05
C18-pos-000269	Isoflav-2-enes (daidzein)	-0.167 ± 0.005	-0.039 ± 0.022	4.72E-08	5.45E-05
C18-pos-007987	α amino acids	-0.141 ± 0.004	-0.064 ± 0.013	2.20E-07	2.12E-04
C18-pos-000858	Amines	0.003 ± 0.009	0.102 ± 0.018	1.50E-06	1.24E-03
C18-pos-001496	Isoflav-2-enes	-0.119 ± 0.007	-0.015 ± 0.021	3.21E-06	2.13E-03
Amide-neg-000894	Isoflav-2-enes (genistein)	-0.017 ± 0.012	0.094 ± 0.019	3.77E-06	2.13E-03
Amide-neg-000144	Flavones (daidzein 4’SO_4)	-0.003 ± 0.009	0.102 ± 0.019	3.77E-06	2.13E-03
C18-pos-007207^^	Benzopyrans (7-HO 4-OCH3 coumarin)	-0.068 ± 0.006	-0.003 ± 0.011	4.06E-06	2.13E-03
C18-pos-004391	Stilbenes	-0.111 ± 0.004	-0.041 ± 0.013	9.65E-06	4.64E-03
C18-pos-009224	Azolines	0.095 ± 0.011	0.045 ± 0.005	1.19E-05	4.92E-03
Hilic-pos-011798	α amino acids	-0.101 ± 0.005	-0.051 ± 0.009	1.24E-05	4.92E-03
Amide-neg-001696	Isoflav-2-enes	-0.014 ± 0.005	0.050 ± 0.012	1.28E-05	4.92E-03
Amide-neg-001204	Isoflav-2-enes (rubiadan)	-0.072 ± 0.006	-0.003 ± 0.014	1.87E-05	6.77E-03
Hilic-pos-022861^^	Androstane steroids	-0.212 ± 0.010	-0.129 ± 0.016	1.99E-05	6.78E-03
C18-pos-022512	Phenol ethers	-0.139 ± 0.005	-0.090 ± 0.009	2.23E-05	7.16E-03
C18-pos-006665^^	Benzopyrans	-0.050 ± 0.006	0.017 ± 0.015	4.33E-05	1.31E-02
C18-pos-021033^^	Benzopyrans	-0.040 ± 0.010	0.037 ± 0.015	4.56E-05	1.31E-02
Hilic-pos-008695**	Keto acid derivatives	-0.055 ± 0.015	-0.127 ± 0.010	4.77E-05	1.31E-02
Hilic-pos-003145**	Tropane alkaloids	0.068 ± 0.011	0.012 ± 0.008	5.44E-05	1.37E-02
C8-pos-003522	Steroid lactones	0.066 ± 0.006	0.130 ± 0.014	5.47E-05	1.37E-02
C18-pos-002347	Quinoline carboxyl acd	-0.089 ± 0.011	-0.003 ± 0.017	6.03E-05	1.41E-02
Amide-neg-011129	α amino acids	-0.242 ± 0.006	-0.186 ± 0.012	6.09E-05	1.41E-02
C8-pos-006275^^	Triterpenoids	0.039 ± 0.006	0.097 ± 0.013	8.61E-05	1.90E-02
Amide-neg-002830	Isoflav-2-enes	-0.070 ± 0.005	-0.010 ± 0.015	8.87E-05	1.90E-02
C18-pos-000980	Amines	-0.017 ± 0.007	0.046 ± 0.014	1.01E-04	2.06E-02
C18-pos-014612^^	Phenylpropanes	-0.034 ± 0.011	0.042 ± 0.013	1.04E-04	2.06E-02
C18-pos-002549	Toluenes	-0.051 ± 0.008	0.011 ± 0.013	1.40E-04	2.70E-02
C18-pos-010657^^	Aryl ketones	-0.083 ± 0.007	-0.036 ± 0.10	1.59E-04	2.96E-02
C18-pos-042082	Benzoxazines	-0.058 ± 0.006	-0.086 ± 0.003	1.90E-04	3.42E-02
C18-pos-002267**	α amino acids	0.184 ± 0.008	0.137 ± 0.010	2.30E-04	4.01E-02
C18-pos-037137**	N-acyl-α amino acids	-0.112 ± 0.010	-0.151 ± 0.006	2.36E-04	4.01E-02
C18-pos-021135**	Benzenesulfonamides	0.029 ± 0.015	-0.037 ± 0.011	2.56E-04	4.23E-02

Among the 35 features identified with differential expression, 23 are not highlighted and also appear in Supplementary Tables 4 and 5, suggesting these differences may have resulted from either protein or carbohydrate consumption. The features indicated by ** were found in greater abundance in children consuming milk protein milk carbohydrate. Those indicated by ^^ were found in greater abundance in children consuming vegetable protein vegetable carbohydrate.

Supplementary Table 7

Comparison of outcomes among Sierra Leonian children with moderate wasting who received supplementary foods with different milk and vegetable protein and carbohydrate content[1]

Characteristic	Milk protein Milk carbohydrate (MPMC) (n = 268)	Milk protein Vegetable carbohydrate (MPVC) (n = 267)	Vegetable protein Milk carbohydrate (VPMC) (n = 272)	Vegetable protein Vegetable carbohydrate (VPVC) (n = 260)	Comparison (95% CI)[2] MPMC vs. VPVC	Comparison (95% CI)[2] MPVC vs. VPVC	Comparison (95% CI)[2] VPMC vs. VPVC
Categorical outcome, n (%)							
Graduate	170 (63)	156 (58)	165 (61)	153 (59)	1.1 (0.9, 1.2)	1.0 (0.9, 1.1)	1.0 (0.9, 1.2)
Remained MAM	30 (11)	30 (11)	29 (11)	27 (10)	1.1 (0.7, 1.8)	1.1 (0.7, 1.8)	1.0 (0.6, 1.7)
Deteriorated to SAM	40 (15)	42 (16)	46 (17)	48 (18)	0.8 (0.6, 1.2)	0.9 (0.6, 1.2)	0.9 (0.6, 1.3)
Death	5 (1.9)	6 (2.2)	2 (0.7)	3 (1.2)	1.6 (0.4, 6.7)	1.9 (0.5, 7.7)	0.6 (0.1, 3.8)
Default	23 (8.6)	33 (12)	30 (11)	29 (11)			
Follow-up visits to graduation; median (IQR)	2 (1, 3)	2 (2, 4)	2 (2, 4)	2 (1, 3)	0.0 (0, 0)	0.0 (0, 1.0)	0.0 (0, 1.0)
Anthropometry[3]							
Rate of weight change, g/kg/d	1.7 (1.5, 2.0)	1.5 (1.3, 1.7)	1.6 (1.3, 1.8)	1.4 (1.2, 1.6)	0.3 (0.0, 0.6)[4]	0.1 (-0.2, 0.4)	0.2 (-0.1, 0.4)
Total weight change, g	455 (407, 504)	439 (391, 487)	457 (404, 510)	392 (336, 447)	64 (-9, 137)	47 (-26, 120)	65 (-7, 138)
Rate of MUAC change, mm/week	1.0 (0.9, 1.2)	0.8 (0.6, 1.0)	0.9 (0.7, 1.0)	0.8 (0.6, 1.0)	0.2 (0, 0.5)	0.0 (-0.3, 0.2)	0 (-0.2, 0.3)
Total MUAC change, mm	4.7 (4.0, 5.4)	4.0 (3.3, 4.8)	4.6 (3.9, 5.4)	3.8 (3.0, 4.6)	0.9 (-0.2, 1.9)	0.2 (-0.9, 1.3)	0.8 (-0.3, 1.8)
Rate of length change, mm/week	1.9 (1.7, 2.1)	2.1 (1.9, 2.3)	2.0 (1.9, 2.2)	2.0 (1.8, 2.2)	-0.1 (-0.4, 0.2)	0.1 (-0.2, 0.4)	0.1 (-0.2, 0.3)
Total length change, mm	11 (9.5, 12)	13 (12, 15)	13 (11, 14)	12 (10, 13)	-0.8 (-2.9, 1.4)	1.6 (-0.6, 3.7)	1.2 (-0.9, 3.4)

[1] Values are mean (95% CI) unless otherwise indicated. Rates of change in anthropometrics were estimated using linear mixed effects models, while total changes in anthropometrics were computed by subtracting the baseline value from the final value. MAM, moderate acute malnutrition; MPMC, milk protein milk carbohydrate; MPVC, milk protein vegetable carbohydrate; MUAC, mid-upper arm circumference; SAM, severe acute malnutrition; VPMC, vegetable protein milk carbohydrate; VPVC, vegetable protein vegetable carbohydrate

[2] Categorical outcomes were compared using modified Poisson regression with robust variance estimates, with estimates over 1 indicating greater relative risk in the milk-containing groups. Continuous outcomes were compared using linear mixed effects models with the exception of the variable "Follow-up visits to graduation" which, because of its skewed distribution and residuals, was compared using Wilcoxon rank-sum test, while the Hodges-Lehmann estimator was used to generate the median of the differences between groups as well as 95% CI. Linear mixed models were specified as follows: Anthropometric measure = Intervention Group * Time + (Time | Participant ID), such that the interaction between group and time (weeks) was modeled as a fixed effect and there were random slopes and intercepts. Estimates over 0 indicate higher values in the milk-containing groups compared with the vegetable-only group.

[3] Several participants defaulted prior to a second visit and so did not have anthropometric changes available for inclusion: MPMC n =9, MPVC n = 13, VPMC n = 8, VPVC n = 8.

[4] $P = 0.030$

Supplementary Figure 1 Enrollment, randomization, and outcomes of study participants

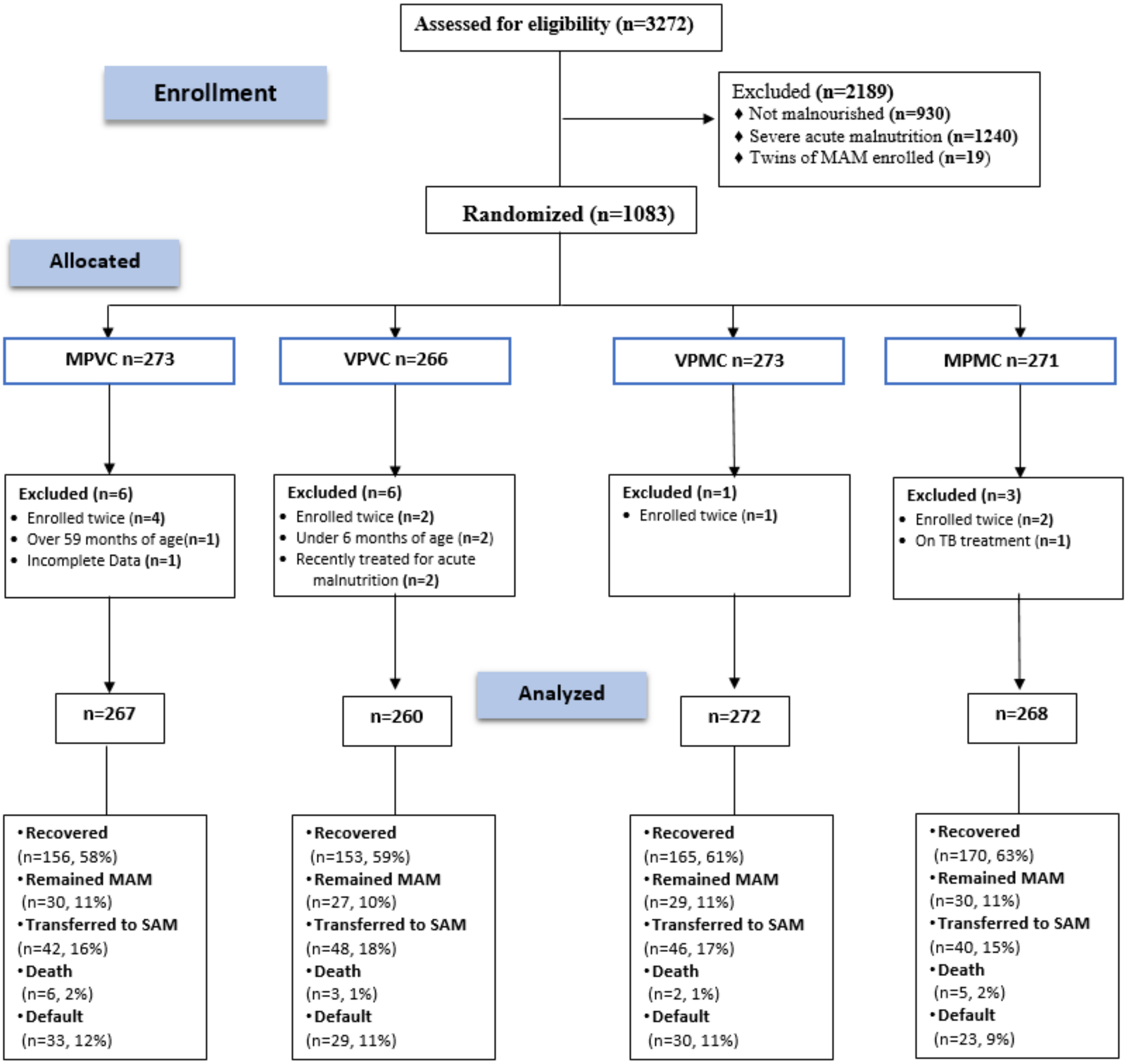

Enrollment, randomization, and outcomes of study participants. Recovered - child reach MUAC ≥ 12.5. Remained MAM - child has not recovered from MAM, after 12 weeks from receiving the first ration of study food (I.e., after receiving 6 biweekly ration of food). Transferred to SAM – child decrees MUAC<11.5, or edema. Default - child missed 3 consecutive clinic visits. Death - child is known to have died. Abbreviations: MPVC- milk protein/vegetable carbohydrate; VPVC – vegetable protein/vegetable carbohydrate; VPMC – vegetable protein/milk carbohydrate; MPMC – milk protein/milk carbohydrate; MUAC, Mid Upper Arm Circumference; SAM, Severe Acute Malnutrition.

Supplementary Figure 2 Change in lactulose excretion from baseline to 4 weeks

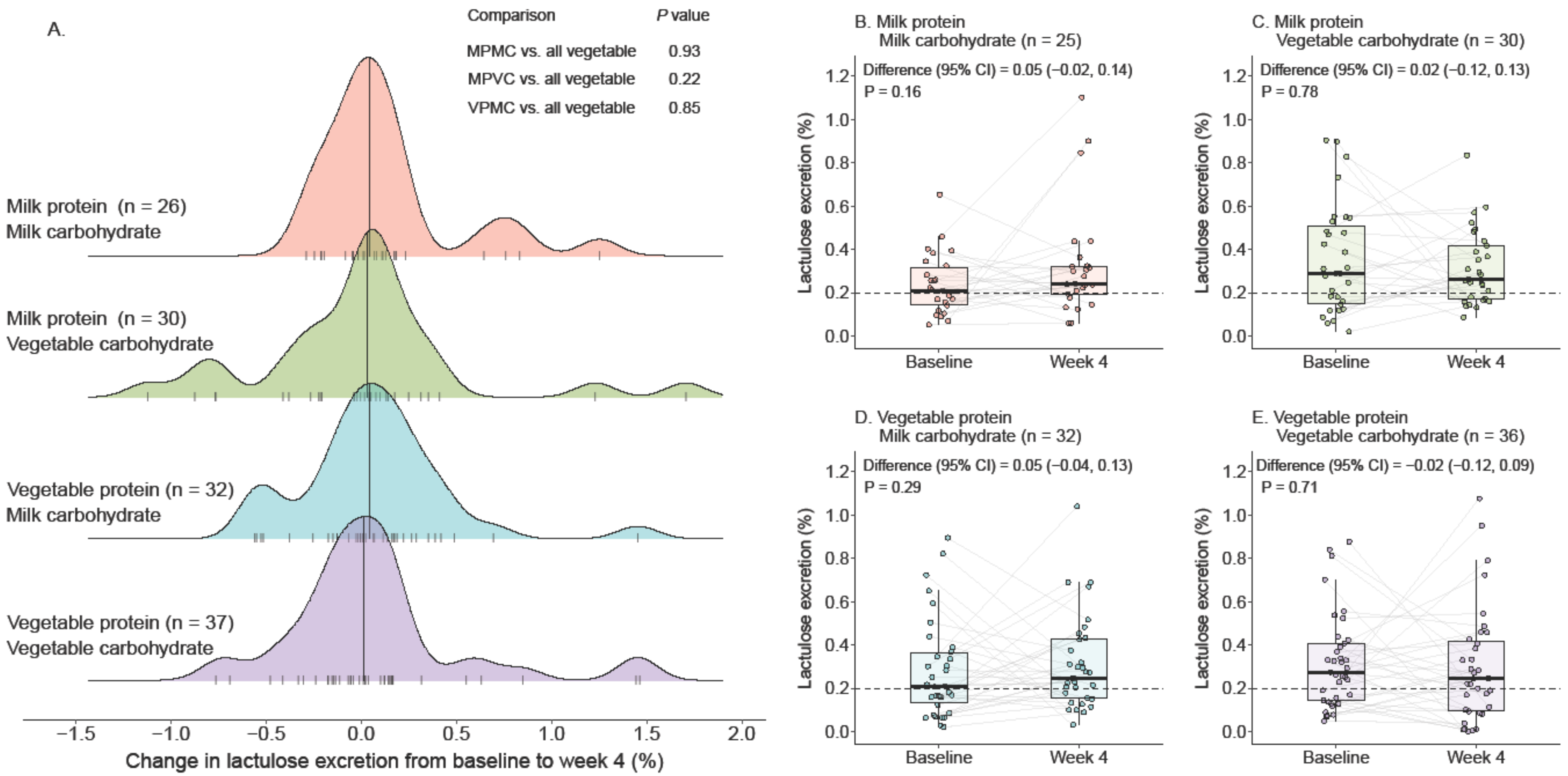

Changes in percent of lactulose excretion among higher risk moderately wasted children (enrollment mid-upper arm circumference < 12.0 cm) in Sierra Leone between baseline and after 4 weeks of supplementary feeding with study foods containing varying milk and vegetable protein and carbohydrate content: milk protein milk carbohydrate (MPMC), milk protein vegetable carbohydrate (MPVC), vegetable protein milk carbohydrate (VPMC), and vegetable protein vegetable carbohydrate (all vegetable). Individuals with measurements at baseline and after 4 weeks of feeding are included. Panel A: ridgeline plots were generated using kernel density estimation. The larger solid vertical line within each distribution represents its median, while the smaller tics beneath each distribution correspond to individual data points. *P* values for each comparison were computed using ordinal logistic regression, with change in % lactulose excretion as the dependent variable, baseline % lactulose excretion as a covariate, and food group as the independent variable of interest. Two measurements were taken after four weeks of supplementary feeding (milk protein / milk carbohydrate n = 1, milk protein / vegetable carbohydrate n = 1). Several measurements were excluded from the visualization due to values > 2% (milk protein / vegetable carbohydrate n = 4, vegetable protein / milk carbohydrate n = 2), but these values were included in all statistical analyses. Panels B – E: spaghetti plots displaying change in lactulose excretion between baseline and week 4. Dots represent the measurements of individual participants, with gray lines connecting an individual's data points. Boxes cover the IQR, the solid line within each box represents the median, and the lines extending from each box cover 95% of values. *P* values for each comparison were estimated using the Wilcoxon signed-rank test, while the Hodges-Lehmann estimator was used to generate the median of the differences between groups as well as 95% CI. The dashed horizontal line within each plot represents a commonly used cut-off between normal (< 0.2) and abnormal (≥ 0.2) % lactulose excretion. To improve visualization, several measurements were excluded from the plots due to values > 1.2% (milk protein / milk carbohydrate n = 1, milk protein / vegetable carbohydrate n = 4, vegetable protein / milk carbohydrate n = 3, vegetable protein / vegetable carbohydrate n = 2).

Supplementary Figure 3 α diversity week 4

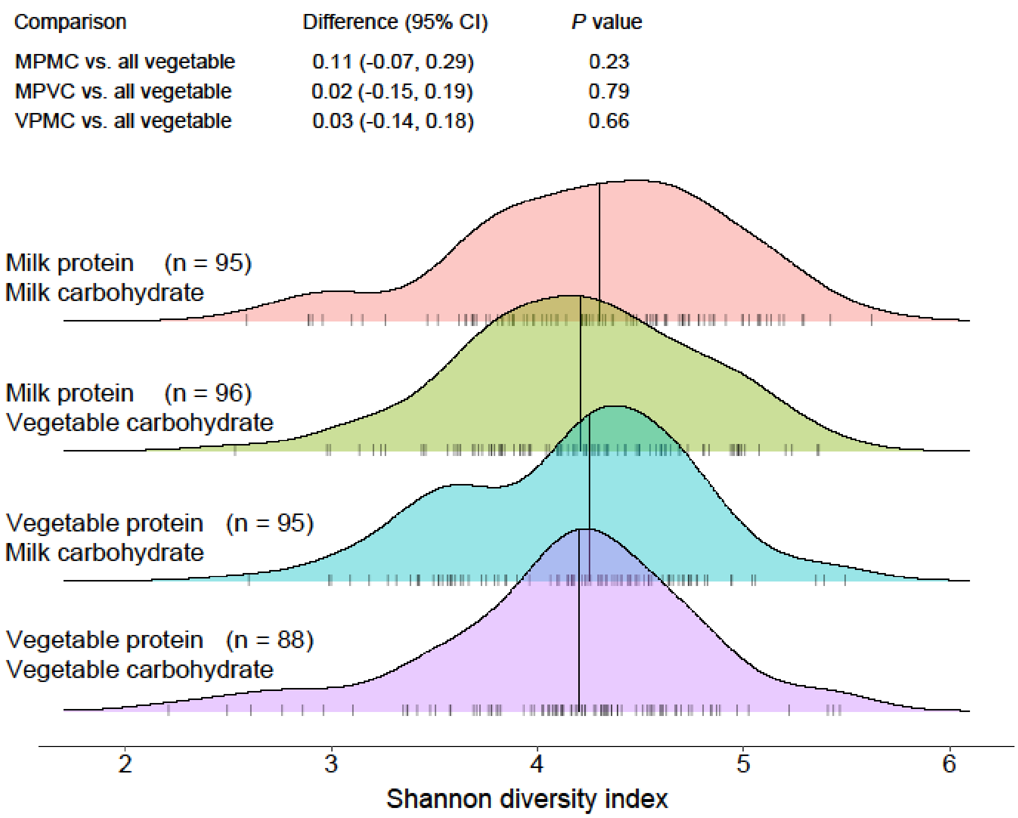

Ridgeline plot of the Shannon α-diversity Index values among higher risk moderately wasted children (enrollment mid-upper arm circumference < 12.0 cm) in Sierra Leone after four weeks of supplementary feeding with study foods containing varying milk and vegetable protein and carbohydrate content: milk protein milk carbohydrate (MPMC), milk protein vegetable carbohydrate (MPVC), vegetable protein milk carbohydrate (VPMC), and vegetable protein vegetable carbohydrate (all vegetable). Ridgeline plots were generated using kernel density estimation. The larger solid vertical line within each distribution represents its median, while the smaller tics beneath each distribution correspond to individual data points. *P* values for each comparison were estimated using the Wilcoxon rank-sum test, while the Hodges-Lehmann estimator was used to generate the median of the differences between groups as well as a 95% CI. Several samples were collected later than 4 weeks due to no-shows or inability of child to pass stool sample on day of study (milk protein milk carbohydrate n = 5, milk protein vegetable carbohydrate n = 6, vegetable protein milk carbohydrate n = 8, vegetable protein vegetable carbohydrate n = 5).

Supplementary Figure 4 Change in α diversity from enrollment to week 4

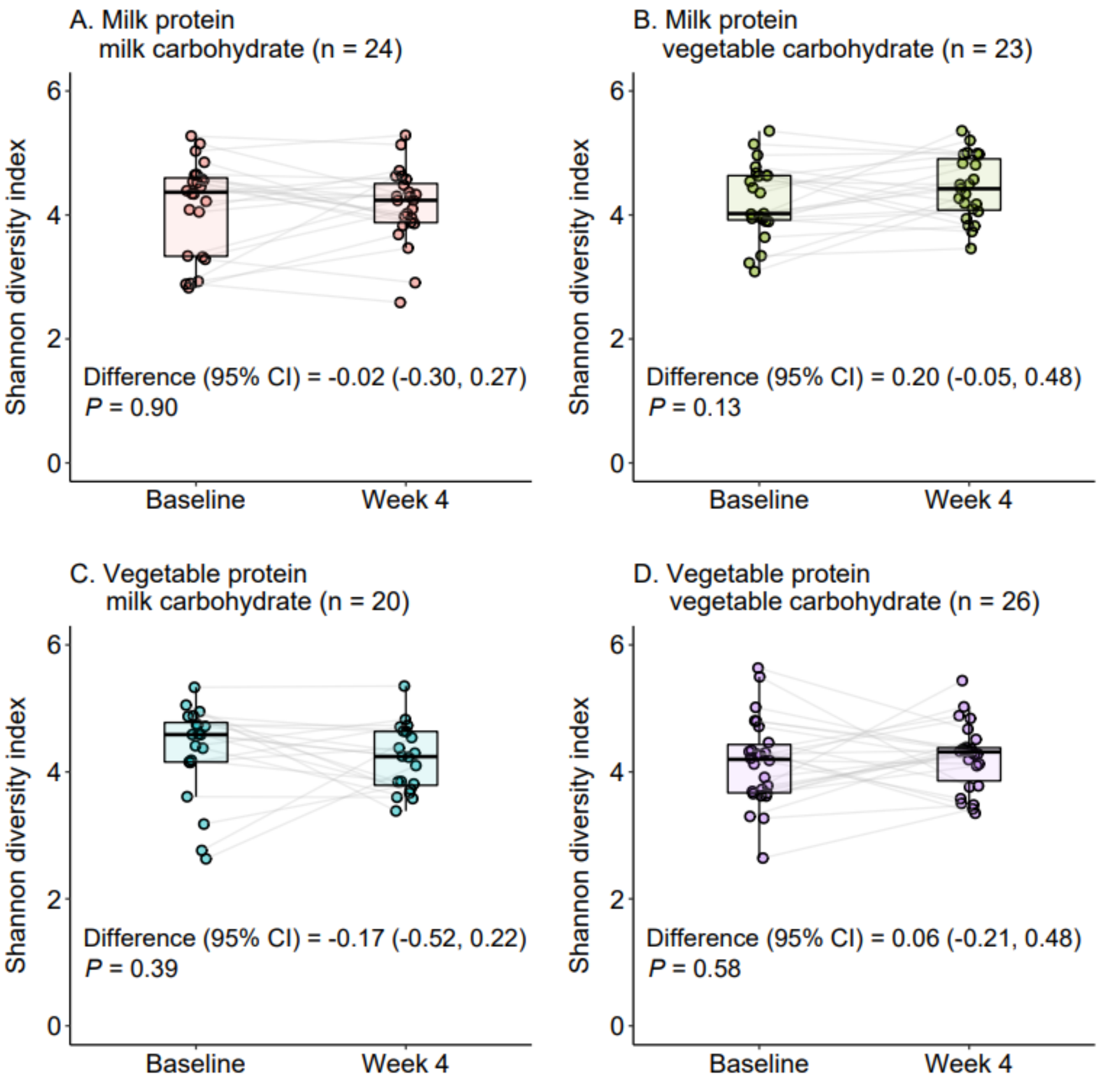

Spaghetti plots displaying change in Shannon α-diversity Index values among higher risk moderately wasted children (enrollment mid-upper arm circumference < 12.0 cm) in Sierra Leone between baseline and after 4 weeks of supplementary feeding with study foods containing varying milk and vegetable protein and carbohydrate content: milk protein milk carbohydrate (MPMC), milk protein vegetable carbohydrate (MPVC), vegetable protein milk carbohydrate (VPMC), and vegetable protein vegetable carbohydrate (all vegetable). Individuals with measurements at baseline and after 4 weeks of feeding are included. Dots represent the measurements of individual participants, with gray lines connecting an individual's data points. Boxes cover the IQR, the solid line within each box represents the median, and the lines extending vertically from each box cover 95% of values. *P* values for each comparison were estimated using the Wilcoxon signed-rank test, while the Hodges-Lehmann estimator was used to generate the median of the differences between groups as well as 95% CI. Several samples were collected later than 4 weeks due to no-shows or inability of child to pass stool sample on day of study (milk protein milk carbohydrate n = 5, milk protein vegetable carbohydrate n = 4, vegetable protein milk carbohydrate n = 6, vegetable protein vegetable carbohydrate n = 3).

Supplementary Figure 5

Most abundant taxa identified by deep 16S sequencing

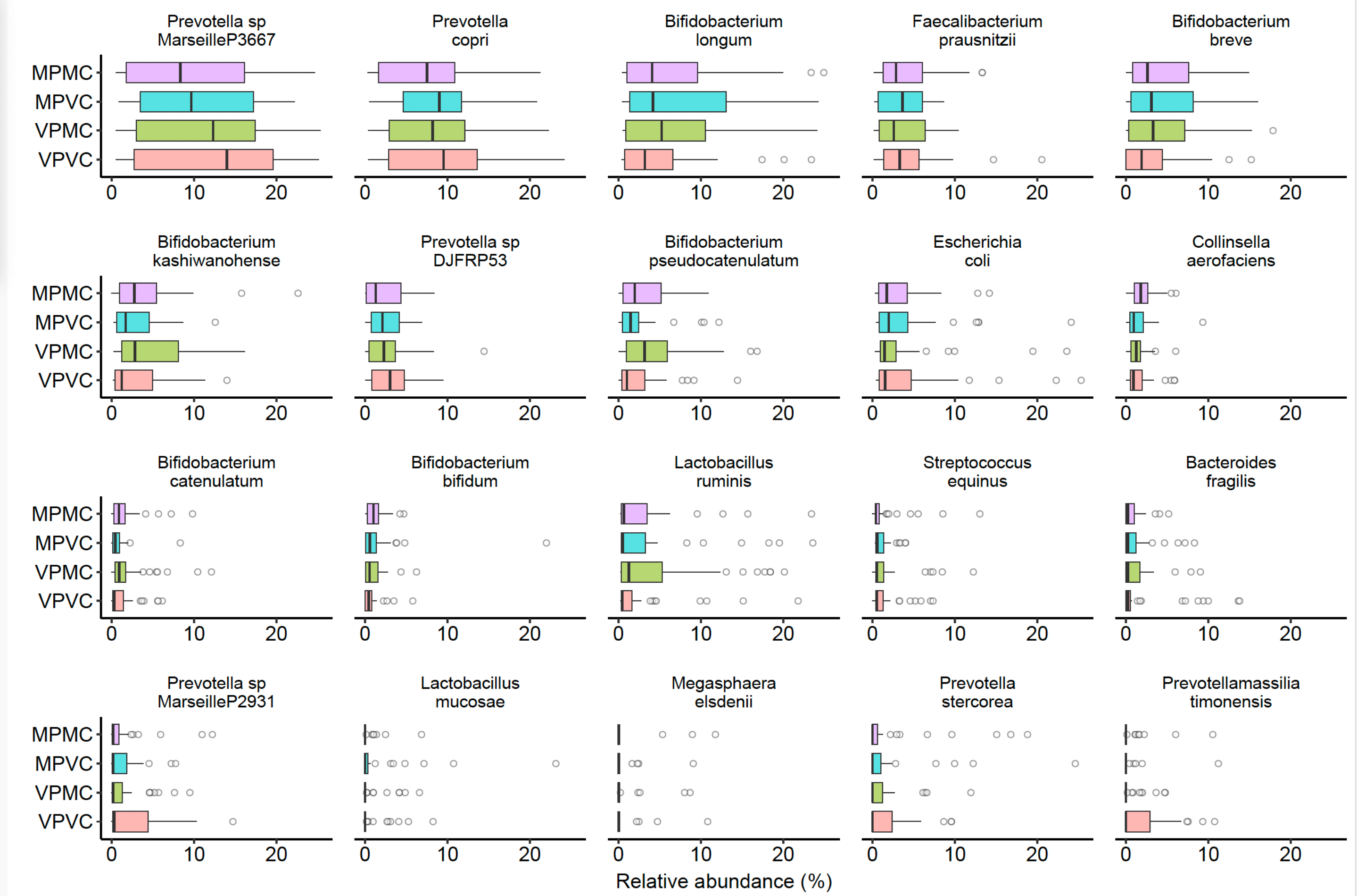

Mean relative abundance of the 20 most abundant taxa identified via deep sequenced 16S rRNA analysis among Sierra Leonean children with high-risk moderate wasting who received 4 weeks of supplementary feeding with study foods containing varying milk and vegetable protein and carbohydrate content: milk protein milk carbohydrate (MPMC, n = 40), milk protein vegetable carbohydrate (MPVC, n = 38), vegetable protein milk carbohydrate (VPMC, n = 47), and vegetable protein vegetable carbohydrate (VPVC, n = 43). The MVRSION pipeline was used to analyze demultiplexed reads from the 7 amplicons covering the nine variable regions to generate a list of microbial species with their corresponding number of reads in each sample. Relative abundances were computed by dividing the absolute abundance of each species by the total species abundance in the sample. Statistical testing was performed on absolute abundance using the Wilicoxon Rank Sum test with a FDR< 0.10 using the Benjamini-Hochberg method.

Supplementary Figure 6

α and β diversity week 4 using only deep sequenced 16S specimens

Comparison	Difference (95% CI)	*P* value
MPMC vs. all vegetable	0.10 (-0.14, 0.32)	0.45
MPVC vs. all vegetable	-0.02 (-0.28, 0.21)	0.85
VPMC vs. all vegetable	-0.03 (-0.27, 0.19)	0.80

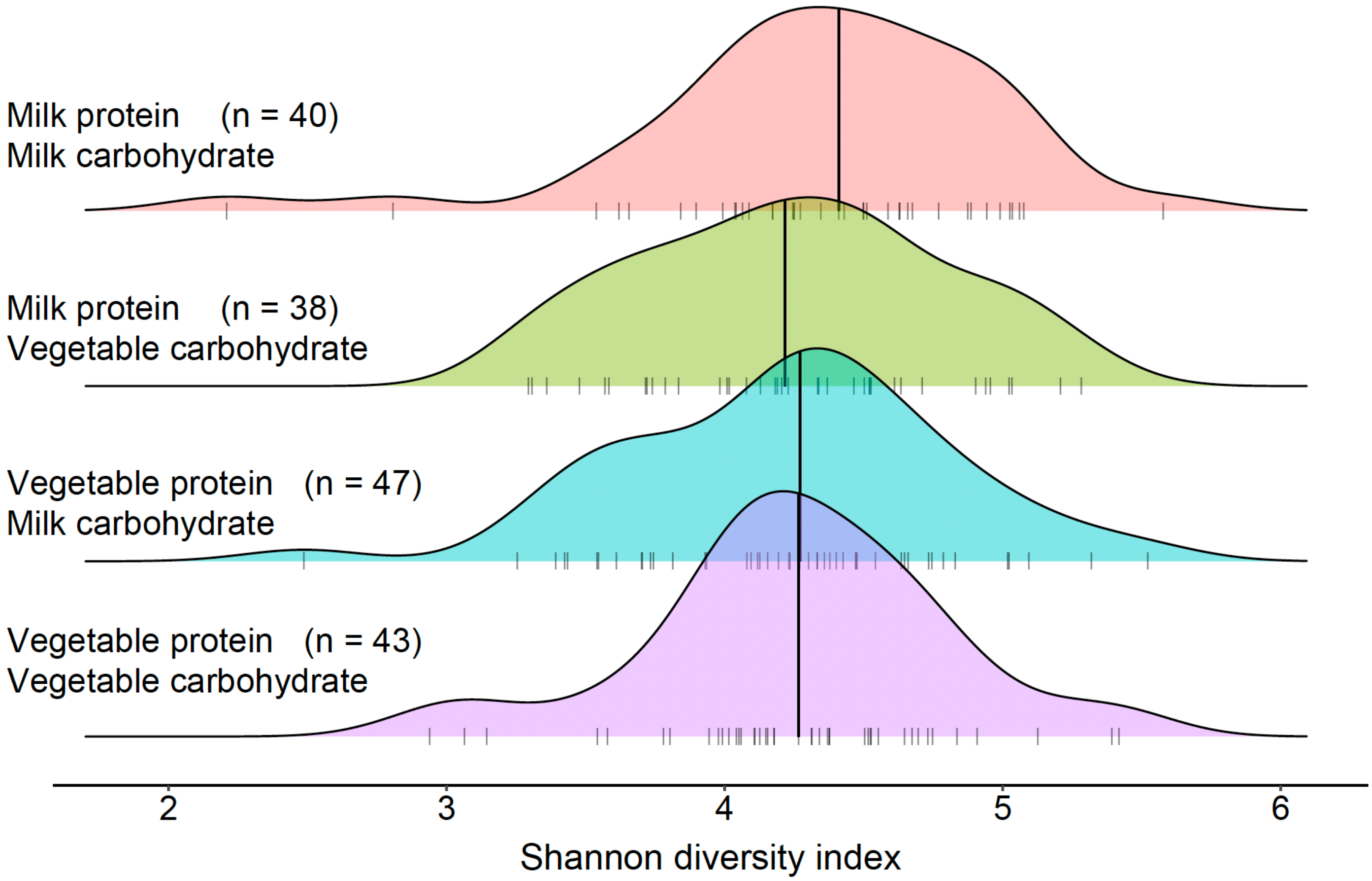

Ridgeline plot of the Shannon α-diversity Index values among higher risk moderately wasted children (enrollment mid-upper arm circumference < 12.0 cm) in Sierra Leone after four weeks of supplementary feeding with study foods containing varying milk and vegetable protein and carbohydrate content. Data used to generate these plots derived only from deep 16S sequencing. Ridgeline plots were generated using kernel density estimation. The larger solid vertical line within each distribution represents its median, while the smaller tics beneath each distribution correspond to individual data points. *P* values for each comparison were estimated using the Wilcoxon rank-sum test, while the Hodges-Lehmann estimator was used to generate the median of the differences between groups as well as a 95% CI.

Weighted Unifrac comparisons between the three supplementary foods containing milk components and the all-vegetable control.

Comparison	Pseudo F statistic	P value
Vegetable protein milk carbohydrate vs VPVC	1.466	0.18
Milk protein vegetable carbohydrate vs VPVC	0.623	0.63
Milk protein milk carbohydrate vs VPVC	1.569	0.18

VPVC, vegetable protein vegetable carbohydrate
